# Btk SH2-kinase interface is critical for allosteric kinase activation and its targeting inhibits B-cell neoplasms

**DOI:** 10.1038/s41467-020-16128-5

**Published:** 2020-05-08

**Authors:** Daniel P. Duarte, Allan J. Lamontanara, Giuseppina La Sala, Sukyo Jeong, Yoo-Kyoung Sohn, Alejandro Panjkovich, Sandrine Georgeon, Tim Kükenshöner, Maria J. Marcaida, Florence Pojer, Marco De Vivo, Dmitri Svergun, Hak-Sung Kim, Matteo Dal Peraro, Oliver Hantschel

**Affiliations:** 1 Swiss Institute for Experimental Cancer Research (ISREC), School of Life Sciences, École polytechnique fédérale de Lausanne (EPFL, 1015 Lausanne, Switzerland; 2 Laboratory of Molecular Modeling and Drug Discovery, Istituto Italiano di Tecnologia, Via Morego 30, 16163 Genoa, Italy; 3 Institute of Bioengineering, École polytechnique fédérale de Lausanne (EPFL), 1015 Lausanne, Switzerland; 4 Department of Biological Sciences, Korea Advanced Institute of Science and Technology (KAIST), Daejeon, Korea; 5 European Molecular Biology Laboratory (EMBL), Hamburg Unit, 22607 Hamburg, Germany; 6 Protein Crystallography Core Facility, School of Life Sciences, École polytechnique fédérale de Lausanne (EPFL), 1015 Lausanne, Switzerland; 7 Institute of Physiological Chemistry, Faculty of Medicine, Philipps-University of Marburg, 35032 Marburg, Germany

**Keywords:** X-ray crystallography, Lymphoma, Target identification

## Abstract

Bruton’s tyrosine kinase (Btk) is critical for B-cell maturation and activation. Btk loss-of-function mutations cause human X-linked agammaglobulinemia (XLA). In contrast, Btk signaling sustains growth of several B-cell neoplasms which may be treated with tyrosine kinase inhibitors (TKIs). Here, we uncovered the structural mechanism by which certain XLA mutations in the SH2 domain strongly perturb Btk activation. Using a combination of molecular dynamics (MD) simulations and small-angle X-ray scattering (SAXS), we discovered an allosteric interface between the SH2 and kinase domain required for Btk activation and to which multiple XLA mutations map. As allosteric interactions provide unique targeting opportunities, we developed an engineered repebody protein binding to the SH2 domain and able to disrupt the SH2-kinase interaction. The repebody prevents activation of wild-type and TKI-resistant Btk, inhibiting Btk-dependent signaling and proliferation of malignant B-cells. Therefore, the SH2-kinase interface is critical for Btk activation and a targetable site for allosteric inhibition.

## Abstract (web summary)

Constitutive Btk signaling drives several B-cell cancers. Here the authors demonstrate key allosteric intramolecular interactions between the SH2 domain and the kinase domain of Btk, and propose an alternative approach for inhibition of both wild-type and tyrosine kinase inhibitor-resistant Btk.

## Introduction

Protein kinases are major drug targets as most cancers carry driver mutations in kinases or are functionally addicted to certain kinase signaling pathways. To date, 55 ATP-competitive kinase inhibitors have been approved for the treatment of multiple solid and hematological tumors^1,2^. A major setback in targeted kinase inhibitor therapy is the development of drug resistance, commonly due to point mutations in the targeted kinase, but also by various other mechanisms^3^. An attractive alternative is the targeting of allosteric sites, other than the ATP binding pocket, critical for the regulation of kinase activity or substrate recruitment. Targetable allosteric regulatory sites have been identified for a few kinases and include the myristoyl binding pocket and SH2-kinase interface in BCR-ABL, as well as the PIF pocket in different AGC kinases^4–7^. As allosteric regulatory pockets are unique to a single kinase or a small class of kinases, one should be able to inhibit oncogenic signaling more selectively. Furthermore, combined or sequential use of allosteric and ATP-competitive inhibitors is a very attractive strategy for cancer treatment, which may diminish or even abolish the outgrowth of resistant clones^5,8^.

Bruton’s tyrosine kinase (Btk) is a central kinase in B-cell receptor (BCR) signaling that is expressed in the B-cell lineage and in myeloid cells. Loss-of-function mutations in Btk are found in humans with X-linked agammaglobulinemia (XLA). These patients are severely immunocompromised due to the impaired development of B-cells^9^. In contrast, elegant functional genomics work has demonstrated that Btk signaling is critical for the survival of the activated B-cell-like (ABC) subtype of diffuse large B-cell lymphoma (DLBCL) and several other B-cell cancers^10,11^. Inhibition of Btk using the FDA-approved BCR-ABL inhibitor dasatinib, which has Btk as one of its major off-targets, provided proof-of-concept evidence for Btk targeting and supported the parallel development of more selective Btk inhibitors^10–12^. Among those, the first-in-class Btk inhibitor ibrutinib^13^, which was approved in 2013, has shown remarkable clinical activity in chronic lymphocytic leukemia (CLL), mantle cell lymphoma (MCL), Waldenström’s macroglobulinemia and graft-versus-host disease. The more selective drugs acalabrutinib^14^ and zanubrutinib^15^ were approved in 2017 and 2019, respectively, as second-line treatments for MCL. However, patients treated with Btk TKIs may acquire resistance caused by Btk mutations of Cys-481 required for covalent binding or, more rarely, by mutations in PLCγ2, downstream of Btk^16^. Therefore, allosteric mechanisms that regulate Btk activity are particularly attractive as additional drug targets to cope with drug resistance in Btk-dependent B-cell malignancies.

Btk and its paralogues Tec, Itk, Bmx and Txk share a conserved SH3-SH2-kinase domain unit with the Src and Abl kinase family of cytoplasmic tyrosine kinases^17,18^. Structural and biochemical data showed that intramolecular interactions of the SH3 and SH2 domains with the kinase domain N- and C-lobe, respectively, result in a compact autoinhibited conformation of Btk analogous to Src and Abl kinases^18,19^. In addition, the N-terminal PH-TH domain module of Btk contributes to stabilizing Btk’s autoinhibited conformation^19,20^. Through inositol phosphate binding to a peripheral site on the PH domain, Btk activation is triggered via dimerization and subsequent trans-autophosphorylation of the kinase domain^19^. Btk activity is positively regulated by two major phosphorylation events. Tyr-551 in the activation loop can be phosphorylated by upstream Src kinases or trans-autophosphorylated by another Btk molecule. Tyr-223, located in the SH3 domain, is the main Btk autophosphorylation site and thought to be autophosphorylated after Tyr-551 phosphorylation^21^. Although an early small-angle X-ray scattering (SAXS) reconstruction suggested a linear and elongated conformation of active Btk^22^, there is little insight on the structural mechanisms and precise molecular events that govern Btk activation.

Here we show that, based on the analysis of XLA mutations in the SH2 domain, Btk activation critically depends on the formation of an allosteric interface between its SH2 and the N-lobe of kinase domain, which we mapped using a combination of enhanced sampling molecular dynamics (MD) simulations and SAXS. Development of a high-affinity engineered protein antagonist to the Btk SH2 domain targeting its interface with the kinase domain prevents Btk activation in cells, inhibits proliferation and Btk-dependent signaling in malignant B-cells. Therefore, we demonstrate the Btk SH2 domain as alternative allosteric site for therapeutic inhibition of Btk and its most common drug-resistant mutant.

## Results

### A set of XLA mutations in the SH2 domain impair Btk kinase activation

**Fig. 1:**
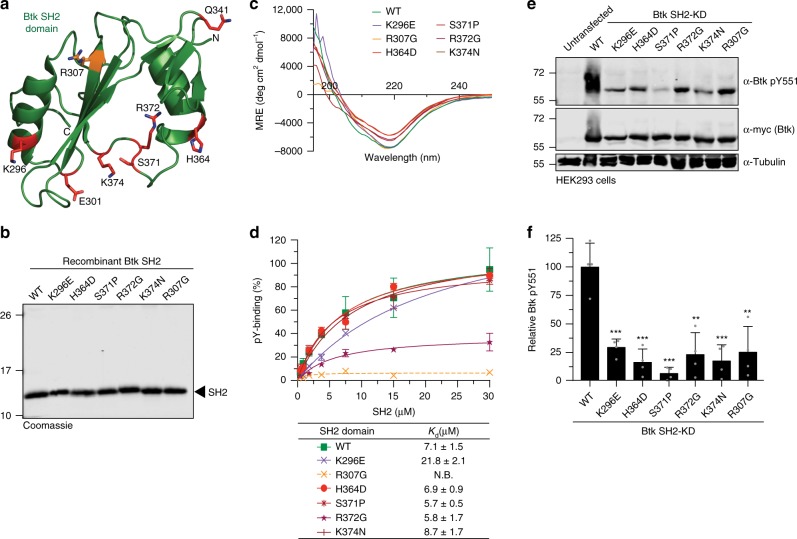
Mutations in Btk SH2 domain abrogates pY551 phosphorylation. **a** Mapping of a subset of XLA-patient mutations (red sticks) onto the human Btk SH2 domain structure (PDB 2GE9). The residue R307 (orange sticks) is part of the pY-binding motif (FIVRD). Here and in all subsequent figures, the residue numbering refers to full-length human Btk. **b** Representative SDS-PAGE analysis of recombinant wild-type and mutant Btk SH2 domains purified from *E. coli*. **c** Averaged far-UV circular dichroism (CD) spectra of recombinant wild-type and XLA mutant Btk SH2 domains. Mean residue ellipticity (MRE) for each protein was calculated from three independent measurements. **d** Binding of a fluorescently labeled pY-peptide to recombinant Btk SH2 domains. Indicated *K*_d_ values were obtained from the fitting to a 1:1 binding model. Data are mean ± SD of two technical replicates. Non-binding (N.B). **e** HEK293 cells were transiently transfected with a construct containing an N-terminal 6xMyc-tagged human Btk SH2-KD wild-type or mutants. Immunoblotting of cell lysates was performed to assess Btk pY551 phosphorylation. **f** Quantification of pY551 immunoblot shown in **e** and normalized to total Btk (Myc) expression. Data are mean ± SD of three biological replicates (*n* = 4) and *P*-values were calculated using an unpaired *t*-test. **P ≤ 0.01 and ***P ≤ 0.001. Source data are provided as a Source Data file.

Sequencing data indicate that approximately 20% of missense mutations in XLA patients are located within the Btk SH2 domain, but how these mutations result in Btk loss-of-function is poorly understood^23^. While several mutations were shown to decrease protein stability and/or impair canonical phosphotyrosine (pY) peptide binding to the SH2 domain^24^, we were intrigued by a mutational hotspot of surface-exposed residues located on the opposite side of the pY-binding pocket and thus unlikely to be involved in pY-peptide binding (Fig. 1a). We first assessed the effects of five representative XLA mutations in this area (K296E, H364D, S371P, R372G, and K374N) and one control XLA mutation (R307G) in the pY binding pocket, on Btk SH2 domain folding, stability, and pY-binding by producing the purified recombinant proteins (Fig. 1b, Supplementary Fig. 1a, b). Far-UV circular dichroism (CD) spectra and thermal shift analysis demonstrated that the XLA mutations did not significantly change Btk SH2 domain folding and only mildly decreased melting temperature compared to the wild-type protein (Fig. 1c and Supplementary Fig. 1c). We next determined the effect of the selected XLA mutations on pY-binding in a fluorescence-polarization (FP) binding assay with a labeled pY-peptide. All XLA mutants bound the pY-peptide with similar affinities as the wild-type Btk SH2 domain, whereas the R307G control mutation in the pY binding pocket strongly impaired binding (Fig. 1d). Thus, the selected XLA mutations do not act by perturbing folding, stability or pY-binding of the Btk SH2 domain.

To determine whether these SH2 mutations affect Btk kinase activity in cells, they were introduced in a Btk SH2-kinase domain (SH2-KD) construct and expressed in HEK293 cells. Expression of wild-type SH2-KD resulted in robust activation loop phosphorylation (pY551; Fig. 1e, f). In contrast, all tested XLA mutations strongly decreased phosphorylation at Y551 (Fig. 1e, f). To test the effect of these mutations on phosphorylation of Y223 within the Btk SH3 domain, which is the most commonly used readout for Btk activity, we introduced them into the larger Btk construct spanning the SH3, SH2 and kinase domains (SH3-SH2-KD). The deleterious effect of an even larger set of XLA mutations in the Btk SH2 domain (Supplementary Fig. 1d) could be corroborated in the SH3-SH2-KD construct with strong impairment of both pY551 and pY223 (Supplementary Fig. 1e, f). Importantly, introduction of a control non-XLA mutation (K311E) on the opposite side of the SH2 domain did not affect pY551 and pY223 (Supplementary Fig. 1e, f). As expected, when the XLA mutants were introduced in the full-length (autoinhibited) protein, no significant effect on pY551 was observed (Supplementary Fig. 1g).

The intriguing observation that certain XLA mutations do not impact on canonical Btk SH2 domain function suggests the presence of a yet unidentified mechanism on how the Btk SH2 domain participates in kinase activation.

### SH2 domain is critical for Btk kinase activation

**Fig. 2:**
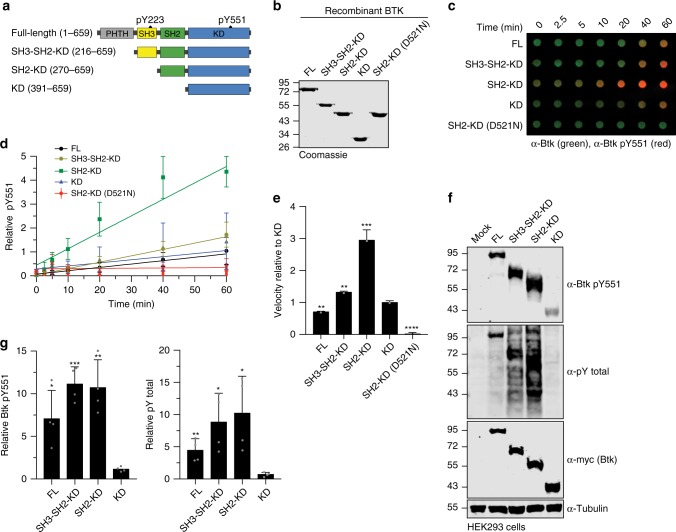
Btk SH2 domain is critical for kinase activation. **a** Schematic representation of Btk constructs used in this study. Construct/domain boundaries and location of the key activating tyrosine phosphorylation sites (pY223 and pY551) are indicated. **b** Representative SDS-PAGE analysis of recombinant untagged Btk proteins purified from Sf9 cells. **c** In vitro autophosphorylation assay performed with recombinant Btk proteins at room temperature. The levels of pY551 (red channel) and total Btk (green channel) were assessed using immunoblotting in a dot blot apparatus. **d** Btk autophosphorylation kinetics shown in **c** normalized to total Btk signal and the calculated slopes of linear fits (relative velocities). Data are mean ± SD of at three independent experiments (*n* = 3). **e** Relative velocities of Btk autophosphorylation relative to KD. Data are mean ± SD of three independent experiments (*n* = 3) and *P*-values were calculated using an unpaired *t*-test. **f** HEK293 cells were transiently transfected with the indicated Btk constructs containing an N-terminal 6xMyc tag and kinase activation assessed by immunoblotting of cell lysates. **g** Quantification of pY551 and total pY shown in **f** normalized to total Btk (Myc) expression and relative to the KD. Data are mean ± SD of three biological replicates (*n* = 4) and *P*-values were calculated using an unpaired *t*-test. *P ≤ 0.05, **P ≤ 0.01, ***P ≤ 0.001 and ****P ≤ 0.0001. Source data are provided as a Source Data file.

As the phenotype of the above-described XLA mutations on Btk kinase activation resembles the phenotype of structure-guided targeted mutations in SH2-kinase domain intramolecular interfaces in the Abl and Fes kinases^6,25^, we hypothesized Btk kinase activation by an analogous allosteric mechanism. To address this, we recombinantly expressed sequential domain deletion constructs (Fig. 2a) in the presence of YopH phosphatase using baculovirus-infected Sf9 cells to obtain unphosphorylated proteins. All proteins were purified to homogeneity (Fig. 2b). Mass spectrometry and immunoblotting analysis confirmed their identity and absence of phosphorylation (Supplementary Fig. 2a, b). These recombinant Btk proteins were incubated with Mg^2+^/ATP and in vitro autophosphorylation on Y551 was monitored over time (Fig. 2c). A kinase-dead Btk SH2-KD protein (D521N) was included as negative control. The SH2-KD construct showed a strong increase in autophosphorylation kinetics compared to the kinase domain alone (KD) and the SH2-KD D521N control (Fig. 2d, e). In agreement with the crystal structure of mouse Btk SH3-SH2-KD, we observed lower pY551 autophosphorylation than with the SH2-KD construct, but still significantly higher than for the Btk KD, as the presence of the SH3 domain likely induced a more closed autoinhibited conformation of Btk, similar to Abl and Src kinases^19^ (Fig. 2d, e). The observed lower in vitro autophosphorylation for full-length Btk (Fig. 2d, e) is in line with a recent molecular model of full-length Btk in solution, where the PH-TH domain docks onto the KD to further stabilize its autoinhibition^20^. Furthermore, strong total pY phosphorylation of SH2-KD was corroborated in this assay (Supplementary Fig. 2c–e) and corresponds to multiple autophosphorylation sites that we mapped using mass spectrometry (MS, Supplementary Table 1). Using this assay, we could also show that phosphorylation on Y223 preceded Y551 phosphorylation in vitro, which agrees with a previous model where autophosphorylation on Y223 may contribute to full activation of the kinase to further transphosphorylate other Btk molecules on Y551^21^ (Supplementary Fig. 2f).

The strong activating effect of the Btk SH2 domain on autophosphorylation in vitro could be corroborated when expressing these constructs in HEK293 cells. Here, the presence of SH2 domain strongly increased Btk autophosphorylation, when compared to KD alone, which shows very low pY551 levels (Fig. 2f, g). Noteworthy, the SH3-SH2-KD construct showed even higher activation when expressed in cells (Fig. 2f, g), which could be due to binding of cellular SH3-SH2 ligands that destabilize the autoinhibited conformation of SH3-SH2-KD. This data indicated that the presence of the Btk SH2 domain is critical for the activation of Btk in vitro and in cells.

### Btk SH2-KD adopts an elongated conformation to trigger kinase activation

We next turned our focus to investigate the structural basis for SH2-dependent allosteric activation of Btk. In contrast to the Fes, Abl and Csk kinases, where SH2-KD units resembling active conformations could be crystallized and had revealed diverse intramolecular interfaces with the N-lobe of the kinase domain, we and others have failed to crystallize active Btk. To provide molecular models of the Btk SH2-KD unit, we used enhanced sampling molecular dynamics (MD) simulations to probe for the interaction of the Btk SH2 domain with the KD. We ran multiple replicas of scaled MD simulations for a total of ~4 µs long trajectories with the Btk SH2-KD unit, including the native SH2-kinase domain linker. Scaled MD is an enhanced sampling MD simulation scheme that allows the sampling of µs-ms time-scale events, such as domain-domain binding^26^. This time frame is prohibitive using classical approaches, such as equilibrium MD simulations. Using this approach, we could sample the binding of SH2 to KD in ~100 ns of simulated time, thus collecting multiple binding events and associated statistics.

**Fig. 3:**
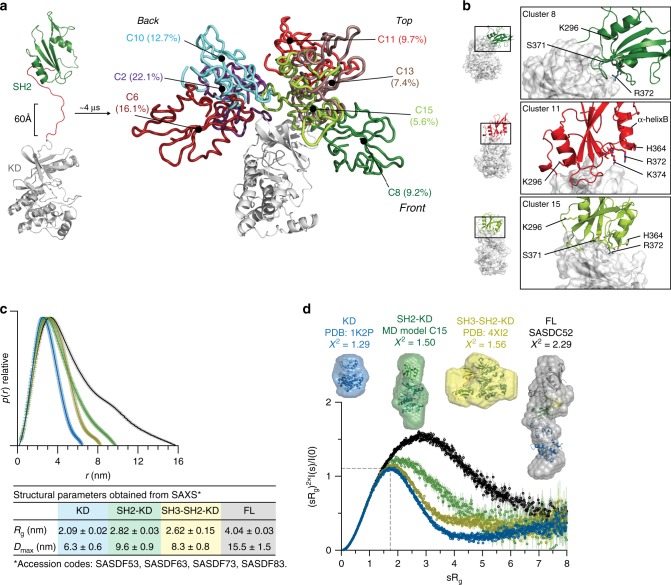
SH2 domain interacts with the N-lobe of the KD in the active Btk. **a** Scaled MD simulations were performed using a model including the Btk SH2 and KD crystal structures (PDB 6HTF and 1K2P, respectively). The most populated clusters of the SH2 positions (several colors) relative to the KD (white) are shown. The percentages indicate the population of the cluster with respect to the entire simulation time. **b** Detailed view of the SH2-KD interface of clusters 8, 11 and 15. SH2 residues mutated in XLA are indicated as sticks. **c** Comparative maximal particle dimension (*D*_max_) of recombinant Btk proteins and summary of structural paraments (*R*_g_ and *D*_max_ ± error) obtained from SAXS. See Supplementary Table 2 for details. **d** Dimensionless Kratky plot of recombinant Btk proteins. The gray dashed line represents the theoretical peak assuming an ideal Guinier region for a globular particle. Ab initio envelope reconstructions obtained from SAXS (surface representation) superimposed on the crystal structures for Btk KD and SH3-SH2-KD (PDB 1K2P and 4XI2, respectively) are shown on top. For the SH2-KD protein, the structure of an elongated MD model with the best agreement with the experimental SAXS data is shown (model C15 shown in **a** and **b**). FL protein shows an extended conformation as observed in a previous SAXS reconstruction (SASDC52). Source data are provided as a Source Data file.

Our MD data demonstrated that the SH2 may interact with the KD at different positions, notably at the back, top, and front of the N-lobe (Supplementary Fig. 3a). The most representative clusters were located in the back of the KD, followed by a more elongated conformation with the SH2 placed on top of the N-lobe (Fig. 3a). Strikingly, the elongated models suggest that several of the SH2 residues mutated in XLA participate in the interaction interface with the N-lobe of the KD (Fig. 3b).

In order to provide an unbiased and independent experimental validation of the MD model, we performed an extensive analysis of multiple recombinant Btk proteins with small-angle X-ray scattering (SAXS). This method allows to directly reconstruct low-resolution particle shapes ab initio and also to study conformational dynamics of multidomain proteins and complexes in solution. From the SAXS data, the SH2-KD construct adopts a more elongated conformation with increased particle dimension (*D*_max_) in comparison to the KD and SH3-SH2-KD proteins (Fig. 3c and Supplementary Fig. 3b). The extended conformation of the SH2-KD protein is independent of kinase activity, as also seen with a kinase-inactive mutant (D521N; Supplementary Table 2). In addition to the increased particle dimension, the normalized Kratky plot suggests that SH2-KD is flexible compared to the globular KD and SH3-SH2-KD proteins (Fig. 3d, bottom). Ab initio shape reconstructions from SAXS corroborate the available KD and (closed autoinhibited) SH3-SH2-KD crystal structures (PDB 1K2P and 4XI2, respectively, Fig. 3d, top*)*. Importantly, the ab initio envelopes of SH2-KD can be superimposed very well with the elongated MD models (e.g., C15, *χ*^2^ = 1.50) in which the SH2 domain is interacting with the N-lobe of the KD (Fig. 3d). The full-length protein has been previously reported to adopt an equilibrium of conformations with a predominant compact and autoinhibited state in solution^20^, whereas our SAXS data from the full-length protein is compatible with its extended conformation and agrees with a previous report^22^. Overall, independent use of MD and SAXS supports an extended model for the Btk SH2-KD, where the SH2 is placed on top and interacts predominantly with the N-lobe of the KD. In line with this model and the data on XLA mutations in the SH2 domain, point mutations in the N-lobe that were structurally predicted to disrupt the allosteric SH2-KD interface impair Btk activation (Supplementary Fig. 3c–e). Interestingly, the L405E mutation in the N-lobe resulted in a strong over-activation of SH2-KD. This gain-of-function phenotype is compatible with an additional salt bridge with positively charges residues in the SH2 domain that may cause a stabilization of the SH2-KD interface (Supplementary Fig. 3f).

**Fig. 4:**
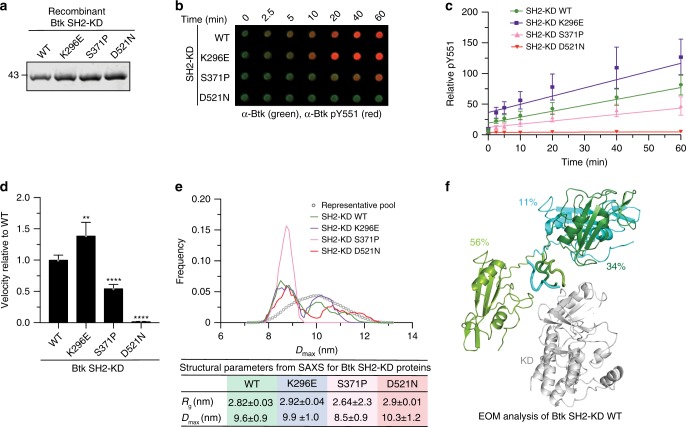
Mutation on the Btk SH2-KD interface perturbs its active conformation and decrease kinase activation. **a** Representative SDS-PAGE analysis of recombinant untagged SH2-KD proteins purified from Sf9 cells. **b** In vitro autophosphorylation assay performed with recombinant Btk proteins at room temperature. The levels of pY551 (red channel) and total Btk (green channel) were assessed using immunoblotting in a dot blot apparatus. **c** Btk autophosphorylation kinetics shown in **b** normalized to total Btk signal and the calculated slopes of linear fits (relative velocities). Data shown are the mean ± SD of two independent experiments (*n* = 6). **d** Relative velocities of Btk autophosphorylation relative to wild-type. Data are mean ±  SD of two independent experiments (*n* = 6) and *P*-values were calculated using an unpaired *t*-test. **P ≤ 0.01 and ****P ≤ 0.0001. **e** Flexibility analysis of SH2-KD based on ensemble of optimization method (EOM) using the experimental SAXS data from recombinant Btk wild-type and mutant proteins. Data from a representative experiment shows the maximal particle dimension (*D*_max_) of selected conformers for each protein (lines) from a representative pool of theoretical conformations (dotted line). The table summarizes the obtained structural parameters (*R*_g_ and *D*_max_ ± error). See Supplementary Table 2 for details. **f** Cartoon representation of the ensemble of SH2-KD conformations selected by EOM analysis (**e**, green line). The percentages represent the contribution of each conformer to a SAXS profile in good agreement with the experimental curve. The KD is represented in white and SH2 conformers are colored. SH2-linker distance estimated by the algorithm is represented as ribbon. Source data are provided as a Source Data file.

To further probe the validity of this model, we took advantage of XLA mutations at different positions on the SH2 surface. Based on our SH2-KD model, S371 is part of the interface with the KD N-lobe, while K296 is solvent-exposed and does not participate in this interdomain interaction (model C15, Fig. 3b). When introduced into Btk SH2-KD and recombinantly purified to homogeneity (Supplementary Fig. 3g, h), the S371P protein showed decreased in vitro autophosphorylation kinetic on Y551 compared to the wild-type protein (Fig. 4a–d). Interestingly, the K296E mutant protein showed a mild increase in Y551 autophosphorylation compared to wild-type, as the mutation may disfavor a more inactive conformation of the SH2-KD protein (Fig. 4a–d). Total pY phosphorylation of the S371P protein was also impaired and further supports the lower autophosphorylation capacity observed for this construct (Supplementary Fig. 3i–k).

To provide additional insights on how these mutations affect SH2-KD conformation, we assessed protein flexibility using SAXS data combined with the ensemble optimization method (EOM^27^). EOM generates a large number of conformations (>10,000) using the KD and SH2 domain structures taking the native linker into account, calculates theoretical SAXS curves for all models, and selects a mixture of conformers that fits the experimental SAXS data. First, SAXS data from the mutants indicated that the S371P protein is somewhat more compact than wild-type, K296E and D521N proteins (Supplementary Fig. 3l, m, Supplementary Table 2). EOM analysis revealed that SH2-KD wild-type is rather flexible with few different conformers co-existing in solution, but indicating extended conformations with the SH2 placed on top of the kinase (Fig. 4e, f). Analysis of the kinase-dead D521N and K296E SH2-KD proteins indicate similar flexibility as for the wild-type protein and also similar overall molecular dimensions (*R*_g_ and *D*_max_). In contrast, the S371P mutant protein showed a shift towards more compact conformation, with a decrease in *R*_g_ and *D*_max_ compared to the wild-type and mutations not affecting the interdomain interface (Fig. 4e). The compact conformation adopted by the SH2-KD S371P is consistent with the decreased phosphorylation observed in the autophosphorylation assay (Fig. 4d). Noteworthy, EOM analysis performed for the SH3-SH2-KD and full-length wild-type proteins is consistent with the models showing, respectively, a compact and a mixture of compact and elongated conformations in solution (Supplementary Fig. 3n). Summarizing, we provide a model for Btk activation via allosteric interaction of the SH2 domain predominantly placed on top of KD. Although the SH2-KD interaction seems less sturdy as in Abl and Fes, a molecular model for the interface of SH2 interacting with the KD can be deduced from our data.

### Development of a protein binder targeting the BTK SH2 domain

**Fig. 5:**
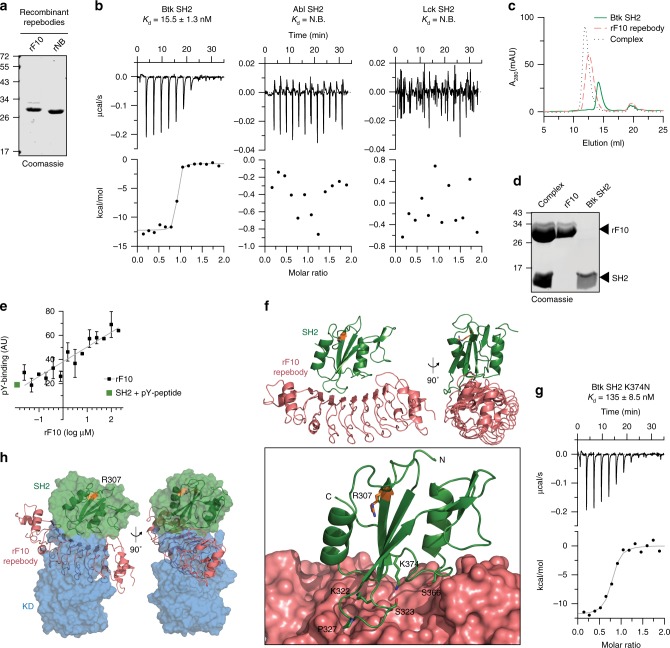
Development of a high-affinity protein binder to the human Btk SH2 domain. **a** Representative SDS-PAGE analysis of recombinant repebodies rF10 and rNB purified from *E. coli*. **b** Representative ITC measurement of rF10 repebody to the SH2 domains of Btk, Abl and Lck kinases. The top panel show the raw signal and the bottom panel show the integrated calorimetric data of the area of each peak. The continuous line shows the best fit to the experimental data assuming a 1:1 binding model. Reported *K*_d_ value for Btk was calculated from three independent measurements. N.B. signifies non-binding. **c** Size-exclusion chromatogram (SEC) analysis of Btk SH2 and rF10 alone, and the SH2-rF10 complex formed by pre-incubation of SH2 and rF10 prior to column injection. **d** Peaks isolated from the SEC analysis shown in **c** resolved by SDS-PAGE and stained with Coomassie. **e** Binding-competition assay using fluorescently labeled pY-peptide to recombinant Btk SH2 domain in the presence of various concentrations of rF10 repebody. Data are mean ± SD from three technical replicates. **f** Cartoon representation of the crystal structure of human Btk SH2 (green) in complex with rF10 repebody (salmon), PDB 6HTF. Structural statistics are reported in Supplementary Table 3. The bottom panel shows the rF10 in surface representation, residue R307 (orange) indicates the position of the pY-binding site, and side chains of SH2 residues interacting with rF10 are shown as green sticks. **g** ITC measurement of rF10 repebody to Btk SH2 K374N performed as in **b**. The *K*_d_ value was calculated from two independent measurements. **h** Superimposition of the active Btk SH2-KD model (surface representation, SH2 in green and KD in blue) with the rF10-SH2 structure (cartoon representation, SH2 in green and rF10 in salmon). Source data are provided as a Source Data file.

In order to demonstrate the importance of the proposed allosteric interaction of the Btk SH2 domain with the kinase domain in regulating Btk activity, we developed a repebody binder. Repebodies are engineered non-antibody scaffold proteins composed of leucine-rich repeat (LRR) modules that can be engineered to bind targets with high specificity. A human Btk SH2 domain-targeting repebody, termed rF10, was generated using phage display selection and affinity maturation^28^ (Supplementary Fig. 4a). rF10 and a non-binding control repebody (rNB) were readily purified from *E. coli* (Fig. 5a). The affinity of rF10 to the Btk SH2 domain is ~15 nM with a binding stoichiometry of 1:1 (Fig. 5b). In contrast, rF10 showed no binding to the SH2 domains from its close relatives, the tyrosine kinases Abl and Lck, demonstrating a >500-fold selectivity for the Btk SH2 domain (Fig. 5b). Consistent with a high-affinity interaction, a stable 1:1 rF10-SH2 complex could be recovered by size-exclusion chromatography either in complex with the Btk SH2 domain alone (Fig. 5c, d) or the full-length Btk protein (Supplementary Fig. 4b, c).

As other engineered SH2 binders, in particular monobodies^29–31^, predominantly target the pY peptide binding site, we first tested whether the rF10 repebody interferes with pY-peptide binding using an FP binding assay. Even in the presence of a 20-fold molar excess of rF10, no competition with pY-peptide binding was observed (Fig. 5e), indicating that rF10 has a different binding epitope on the SH2 domain. The observed increased FP signal with increasing rF10 concentrations is consistent with the formation of a ternary complex (SH2-pY-peptide-rF10; Fig. 5e).

We next solved the crystal structure of the Btk SH2-rF10 complex at 1.9 Å resolution (Fig. 5f, Supplementary Table 3, PDB 6HTF), which is the first crystal structure of the isolated human Btk SH2 domain. The overall SH2 domain conformation is very similar to the previously published NMR structure of human Btk SH2 domain^32^ (PDB 2GE9), indicating that rF10 binding does not result in major conformational changes^31,33^. Consistent with the ITC and pY binding assays, the rF10-SH2 crystal structure indicates that rF10 binds to SH2 domain in a 1:1 interaction and the interaction does not involve the pY-binding groove. rF10 binds to multiple residues from the SH2 domain BC loop (K322, S323, G325 and P327) and the C-terminus of the α-helix B (S366 and K374), and it buries a surface area of 2274 Å^2^ (Fig. 5f). To further corroborate the rF10 interaction site, a recombinant SH2 containing the XLA mutation K374N in the interface between the SH2-rF10 showed a ~9-fold decreased affinity (Fig. 5g) compared to the wild-type SH2 domain.

Superimposition of the Btk SH2-rF10 structure with a representative SH2-KD structure (MD model C15) revealed dramatic steric clashes of the KD and rF10 (Fig. 5h, Supplementary Movie 1). The coincidental strong overlap of the rF10 binding epitope with the proposed Btk SH2-kinase interface led us to hypothesize that rF10 may abrogate the SH2-KD interaction and thereby act as an allosteric Btk antagonist. To probe this hypothesis, we first performed SAXS analysis of rF10 alone and in complex with several Btk constructs (Supplementary Fig. 4d, e, Supplementary Table 4). In line with our hypothesis, SAXS-based reconstructions of rF10-Btk complexes suggests that the conformation of SH2-KD is altered and the interdomain interface is disrupted (Supplementary Fig. 4f). This observation encouraged us to further investigate the functional effects of the rF10 repebody on Btk activity and signaling in vitro and in cells.

### Targeting the SH2-KD interface with rF10 inhibits Btk kinase

**Fig. 6:**
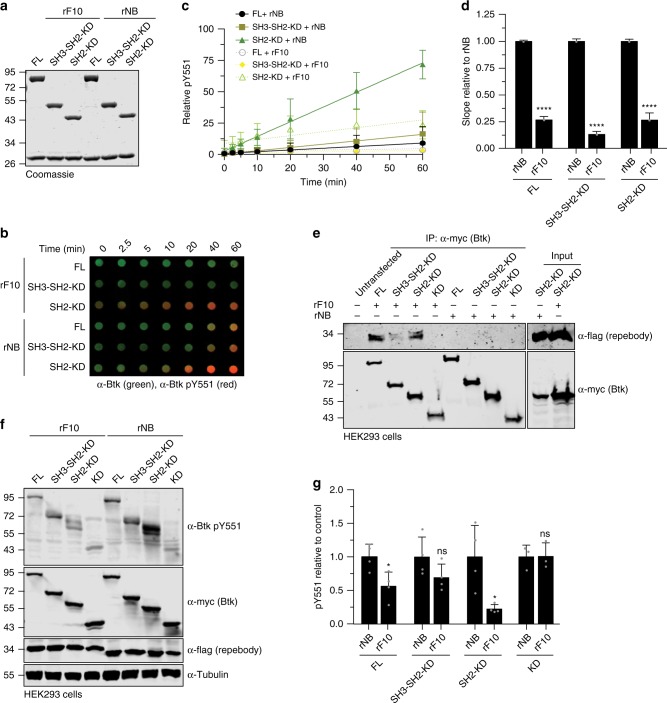
rF10 repebody inhibits Btk activation in vitro and in cells. **a** Representative SDS-PAGE analysis of in vitro autophosphorylation assay containing recombinant Btk proteins mixed with rF10 or non-binding (rNB) control repebodies. **b** In vitro autophosphorylation assay performed with recombinant Btk protein with 2-fold excess of rF10 or rNB at room temperature. The levels of pY551 (red channel) and total Btk (green channel) were assessed using immunoblotting in a dot blot apparatus. **c** Btk autophosphorylation kinetics in the presence of rF10 (dashed lines) or rNB (continuous lines) repebodies shown in **b** normalized to total Btk signal and the calculated slopes of linear fits (relative velocities). Data are mean ± SD of three independent experiments (*n* = 6). **d** Relative velocities of Btk autophosphorylation relative to control repebody. Data are mean ± SD of three independent experiments (*n* = 3) and *P*-values relative to rNB were calculated using an unpaired *t*-test. **e** HEK293 cells were transiently co-transfected with indicated Btk constructs and repebodies, and cell lysates subjected to immunoprecipitation using anti-Myc coated beads. A representative sample of cell lysate for each repebody is shown as expression control. **f** Immunoblot analysis of cell lysates from HEK293 cells transiently co-transfected with indicated Btk constructs and repebodies used to assess Btk pY551 phosphorylation. **g** Quantification of pY551 shown in **f** normalized to total Btk (Myc) expression and relative to rNB control. Data are mean ± SD of three biological replicates (*n* = 4) and *P*-values were calculated using an unpaired *t*-test. *P ≤ 0.05, ****P ≤ 0.0001, and non-significant (ns). Source data are provided as a Source Data file.

As rF10 was confirmed to bind the Btk SH2 domain, we first measured the binding affinity of rF10 to recombinant SH2-KD, SH3-SH2-KD and full-length Btk. rF10 was found to bind all three proteins with similar low nanomolar affinities (Supplementary Fig. 5a). We next performed in vitro autophosphorylation assays using different recombinant Btk constructs in the presence of a 2-fold molar excess of rF10 or rNB control (Fig. 6a, b). rF10 showed a strong inhibitory effect on pY551 autophosphorylation of all tested Btk constructs containing the SH2 domain (full-length, SH3-SH2-KD and SH2-KD; Fig. 6c, d). Interestingly, even though the constructs SH3-SH2-KD and full-length Btk adopt an autoinhibited conformation with low autophosphorylation activity (Fig. 2c–e), rF10 strongly decreased their remaining activity (Fig. 6c, d). Consistent with this data also total pY phosphorylation of Btk was decreased in the presence of rF10 (Supplementary Fig. 5b–e).

To test the ability of rF10 to act an allosteric Btk inhibitor in cells, we first tested whether rF10 interacted with different Btk constructs in mammalian cells. Pull-down assays showed interactions of rF10, but not of the rNB control repebody, with all Btk constructs containing the SH2 domain, but not with the Btk KD alone (Fig. 6e). In the presence of rF10, lower levels of Btk pY551 and total pY were observed than when equal amounts of rNB were expressed. Phosphorylation of the KD alone was unaltered in the presence of repebodies, indicating selective SH2 domain-dependent inhibition of Btk autophosphorylation by rF10 (Fig. 6f, g and Supplementary Fig. 5f, g). To determine the effect of allosteric inhibition of Btk by rF10 on kinase activity, we performed in vitro kinase assays with a substrate peptide encompassing Tyr-753 of PLCγ2, a canonical Btk substrate. In the presence of rF10, but not rNB, kinase activity of full-length Btk was strongly inhibited (Supplementary Fig. 5h).

This data collectively showed that targeting the Btk SH2 with a repebody binder at the proposed SH2-kinase interface selectively inhibits Btk activity.

### rF10 decreases viability and inhibits Btk signaling of lymphoma cells

**Fig. 7:**
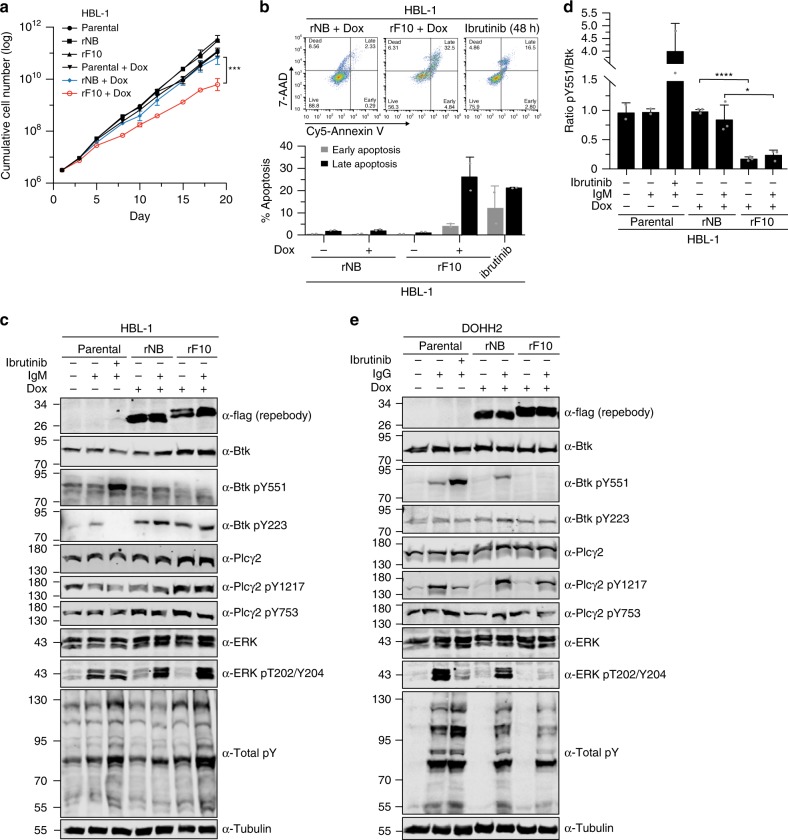
Targeting the Btk SH2-KD interface decreases the viability of B-cell lymphoma cells and inhibits BCR signaling. **a** Cumulative cell number of HBL-1 cells lentivirally transduced with a doxycycline-inducible system for expression of rF10 or rNB control repebodies (*n* = 4). Parental cells are non-transduced cells. **b** Apoptosis analysis of transduced HBL-1 expressing the repebodies for 7 days. A representative gating of FACS staining is shown on top. The quantification of early (7AAD-/Annexin V+) and late (7AAD+/Annexin V+) apoptotic cells was obtained from two independent experiments (*n* = 2). Parental HBL-1 cells treated with 10 µM of ibrutinib for 48 h were used as positive control. **c** Immunoblot analysis from transduced HBL-1 cells expressing the repebodies (Flag-tagged) for 48 h. BCR signaling was stimulated with anti-human IgM or mock-treated for 2 min before cell lysis. Ibrutinib treatment (100 nM) was performed for 15 min prior to anti-IgM stimulation. Tubulin was used as loading control. **d** Quantification of Btk pY551 shown in **c** and normalized to total Btk expression. Data are mean ± SD from two biological replicates (*n* = 3) and *P*-values were calculated using an unpaired *t*-test. *P ≤ 0.05. **e** Immunoblotting from transduced DOHH2 cells expressing the repebodies as performed in **c**. Source data are provided as a Source Data file.

Finally, we investigated whether targeting the SH2-KD interface is sufficient to inhibit Btk activity in neoplastic B-cells. We selected DLBCL cell lines that express wild-type Btk and are sensitive to ibrutinib (Supplementary Fig. 6a) and transduced them to inducibly express rF10 or the rNB. Upon induction of rF10 expression by doxycycline in HBL-1 cells, we observed a more than 10-fold reduction in cumulative cell numbers as compared to rNB, all uninduced conditions or parental cells (Fig. 7a). This was accompanied by a dramatic increase in apoptosis, comparable to the treatment of parental cells with ibrutinib (Fig. 7b). Also in TMD8 cells, a decrease of the cumulative cell numbers were observed (Supplementary Fig. 6b). Cells in which the SH2-kinase interface is targeted with rF10 remain sensitive to ibrutinib (Supplementary Fig. 6c). Combined rF10 expression and ibrutinib treatment further increased apoptosis, as compared to the single perturbations (Supplementary Fig. 6d). These results indicated that allosteric inhibition of Btk represents a second targeting mechanism and that combinations with ATP-competitive drugs might be beneficial.

rF10 expression promotes decreased Btk pY551 phosphorylation in HBL-1 cells, which could not be recovered by BCR stimulation using anti-human IgM (Fig. 7c, d). Interestingly, an increase in Btk and PLCγ2 protein levels was observed upon rF10 expression, which may suggest a compensatory mechanism to counteract the activity of rF10 on Btk inhibition (Fig. 7c and Supplementary Fig. 6e). The rF10 effects on BCR signaling were also consistent in DOHH2 cells. Here, rF10 expression resulted in decreased Btk pY551, even upon BCR stimulation, decreased PLCγ2 phosphorylation on Y1217, one of the two main Btk phosphorylation sites, as well as strongly decreased in Erk activation (Fig. 7e).

**Fig. 8:**
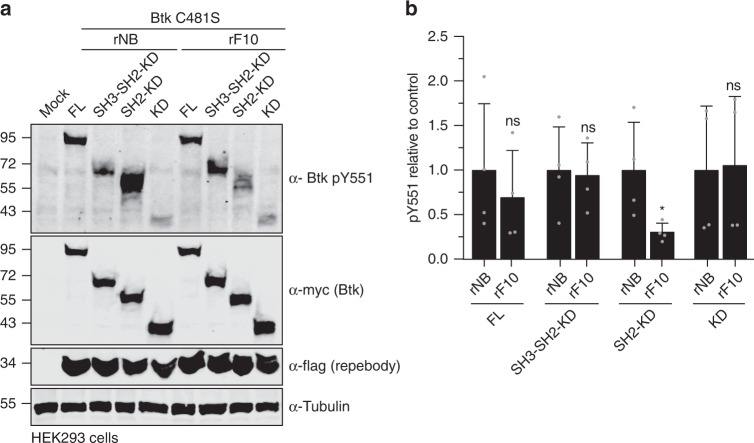
Targeting the Btk SH2-KD interface decreases activation of therapy-resistant Btk with mutation on C481. **a** Immunoblot analysis of cell lysates from HEK293 cells transiently co-transfected with indicated Btk C481S constructs and repebodies used to assess Btk pY551 phosphorylation. **b** Quantification of pY551 shown in **a** normalized to total Btk (Myc) expression and relative to rNB control. Data are mean ± SD of two biological replicates (*n* = 3) and *P*-values were calculated using an unpaired *t*-test. *P ≤ 0.05. Source data are provided as a Source Data file.

We next tested whether targeting the SH2-KD interface offers an alternative approach to target TKI-resistant Btk. Importantly, rF10 was able to decrease pY551 (Fig. 8a, b) and total pY (Supplementary Fig. 7a, b) of Btk C481S SH2-KD expressed in HEK293 cells and to a similar extent than of wild-type SH2-KD.

To our knowledge, this is the first report of an alternative mechanism able to inhibit wild-type and drug-resistant Btk by targeting an allosteric site. Together, we demonstrated that the SH2-KD interface is critical for Btk kinase activation and a targetable site to improve therapies of Btk-driven malignancies.

## Discussion

While recent studies provided considerable insight on how Btk is autoinhibited, little is known about the interdomain rearrangements coordinating Btk activation^18^. Here, we demonstrated that an unsuspected allosteric interaction between the Btk SH2 and KD is critical for kinase activation thereby explaining the loss-of-function phenotype of a subset of XLA mutations in the SH2 domain. Furthermore, we identified and validated the SH2-KD interface as a unique site for allosteric Btk inhibition using an engineered repebody protein. To our knowledge, this is the first alternative targeting strategy for ibrutinib-resistant Btk variants. The three FDA-approved Btk inhibitors, ibrutinib, acalabrutinib and zanubrutinib are highly susceptible to resistance development by mutations of C481 to which these inhibitors covalently bind. Therefore, the therapeutic exploitation of the Btk SH2-KD interface for patients with TKI-resistant B-cell malignancies is highly attractive.

SH2 domains are conserved, abundant domains in proteomes, and hence difficult to target using small molecules. Small engineered protein binders, in particular monobodies, have emerged as powerful tools to target SH2 domains of a variety of kinases and phosphatases and encourage the development of alternative SH2 inhibitors^30,31,33^. In particular, recent advances in cytoplasmic delivery strategies of protein binders, its combination with targeted protein degradation and feasibility PROTAC-based Btk degradation demonstrate progress towards therapeutic applicability^34,35^. The combined targeting of different sites on the same Btk molecule to limit resistance development is likely to follow the paradigm of the allosteric Bcr-Abl myristoyl pocket inhibitor asciminib, which abrogates drug resistance when combined with ATP-competitive Bcr-Abl inhibitors^36^, In addition to the SH2-kinase interaction, inhibition of dimerization of the PH-TH domain, which is required for membrane-associated activation of Btk, was proposed for allosteric targeting although not yet explored^19^.

It is important to note that targeting the Btk SH2-KD interface leads to particularly strong effects in DLBCL cell lines harboring CD79B ITAM mutations (Y196F in HBL-1 and Y196H in TMD8 cells) that trigger chronic activation of BCR, and are therefore heavily dependent on Btk signaling to support cell growth. This may indicate that patients with this genotype might benefit strongly from allosteric targeting approaches of Btk^10^. Besides B-cell malignancies, Btk is an emerging target in autoimmune diseases, e.g. rheumatoid arthritis. Here, allosteric inhibition of Btk in Fc receptor (FcRs) signaling in basophils and mast cells could provide potential therapeutic benefits^37^.

Previous studies on other Tec kinase members, in particular Itk, reported an increase in kinase activity in the presence of protein-interacting domains, including the SH2 domain^38,39^. This early mechanistically unexplained observation may hint towards conservation of the allosteric SH2-kinase interface in all Tec kinases. Strikingly, residues involved in the Btk SH2-kinase interface are identical in most other Tec members, but are not conserved in the Abl and Fes kinase families and residues critical for the Abl SH2-kinase interaction are not conserved in Tec kinases^6,25^ (Supplementary Fig. 7c). This further supports the notion that allosteric SH2-kinase interfaces in different kinase families appear to be diverse in terms of location, charge, hydrophobicity, size, and, importantly, dynamics. The Btk SH2-KD interaction seems highly dynamic, which precluded crystallization, in contrast to the respective Fes and Abl SH2-KD constructs^25,40^, as well as Csk, where both SH2 and SH3 domains contact the N-lobe^41^.

Our data also provides a first structural insight on the molecular mechanism-of-action of several SH2 mutations in XLA. Those may disturb Btk activity by shifting the SH2-KD conformation towards a more compact and thus less active kinase compared to a native elongated conformation. Additional multi-domain structures including the SH2-kinase linker will potentially capture the preferential position of the SH2 towards the KD, and further corroborate the model of SH2-mediated activation of Tec kinase members.

Our study adds important structural insights into the complex regulation of Tec kinases, where the SH2 domain plays a critical role in kinase activation, which is independent of its canonical function. Disruption of the SH2-KD interface hampers Btk activation and provides a molecular mechanism that explains a subset of pathogenic XLA mutations. Finally, the exploration of the SH2-KD interface as a targetable allosteric site, even in therapy-resistant Btk variants, provides an attractive approach to target Btk and potentially other Tec kinases.

## Methods

### Cell lines and culture conditions

HEK293 (ATCC, CRL-1573) and HEK293T (ATCC, CRL-3216) cells were cultured in DMEM (Gibco) supplemented with 10% fetal calf serum (Gibco) and 1% penicillin/streptomycin (Bioconcept). Human DLBCL cell lines DOHH2, HBL-1 and TMD8 (expressing full-length wild-type Btk protein) were kindly provided by M. Thome-Miazza (University of Lausanne), cultured in RPMI-1640 media (Gibco) supplemented with 1 mM L-glutamine (Gibco), 10% fetal calf serum (Gibco) and 1% penicillin/streptomycin (Bioconcept). All cell lines were cultured at 37 °C under 5% CO_2_.

### Protein expression and purification

Btk SH2 domains were cloned into the pETM30 plasmid with an N-terminal 6xHis-GST tag with tobacco etch virus (TEV) cleavage site, and expressed in *E. coli* Bl21(DE3). Repebodies were cloned into the pET21a plasmid (Millipore) with a C-terminal 6xHis-tag, and expressed in *E. coli* Origami (DE3). Expression of recombinant proteins was performed overnight at 18 °C in LB medium after induction with 0.5 mM IPTG at an optical density of ~0.8. For protein purification, bacteria were harvested in purification buffer (50 mM Tris pH 7.5, 500 mM NaCl, 1 mM DTT, 5% glycerol, 10 mM imidazole) containing DNase, homogenized using an Avestin Emulsiflex C3 homogenizer, followed by lysate clarification through centrifugation. Proteins were first purified by gravity flow Ni-NTA agarose (Qiagen, 30210) followed by tag cleavage with recombinant TEV protease in dialysis in buffer (25 mM Tris pH 7.5, 300 mM NaCl, 1 mM DTT, 5% glycerol). Finally, samples were subjected to size exclusion chromatography (SEC) on a Superdex 75 16/60 column equilibrated with dialysis buffer, and peak fractions pooled and analyzed by SDS-PAGE.

For insect cell expression, sequences were cloned into a pFast-Bac-Dual plasmid (Thermofisher). To obtain unphosphorylated Btk, Flag-tagged Yersinia protein tyrosine phosphatase (YopH) was simultaneously expressed from the same vector. Baculoviruses were prepared following the instructions from Bac-to-Bac Baculovirus Expression System (Thermofisher) protocol. Briefly, pFast-Bac Dual plasmids were transfected to *E. coli* DH10B followed by bacmids purification (PureLink, Invitrogen) and transfection in Sf9 cells using the transfection reagent FuGene HD (Promega, E2311). Supernatant containing the baculoviruses were used to produce recombinant proteins in Sf9 cells at density 1.5 × 10^6^ cells mL^−1^ in SF-900 SFM (10902-096, Thermo) cultured at 28 °C and 80% air humidity. After 3 days, cells were and resuspended in purification buffer containing 1 mM PMSF, protease cocktail inhibitor (Roche) and Benzonase (Millipore), and lysed by sonication. Cleared lysates were purified and tags removed as described above. All purified proteins could be stored at −80 °C without loss of activity. Absence of YopH phosphatase activity was reassured by a phosphatase activity assay using colorimetric PNPP substrate (Thermo) and immunoblotting.

### Site-directed mutagenesis

All point mutations were introduced using the Quikchange II Site-Directed Mutagenesis Kit (Agilent) using primers described in Supplementary Table 5. Sequence alignments were generated using Geneious (Biomatters).

### Kinase autophosphorylation assay

1 µM of recombinant Btk proteins were incubated in Tris 25 mM pH 7.5, 150 mM NaCl, 5% glycerol, 1 mM ATP, 20 mM MgCl_2_, 1 mM DTT. For inhibition of autophosphorylation, 2 µM of repebodies were pre-incubated with 1 µM of Btk proteins for 15 minutes before starting the reaction upon the addition of 1 mM ATP. Reactions were carried out at room temperature and stopped at desired time points by adding 2X Laemmli buffer to each tube, followed by boiling 5 minutes at 95˚C. Samples were immunoblotted onto a nitrocellulose membrane using a Dot-Blot apparatus (Bio-Rad).

### HEK293 transfection

Btk constructs were expressed in HEK293 cells using pCS2-gateway plasmid containing an N-terminal 6xMyc tag, while repebodies were cloned into pcDNA3.1 vector and contained a C-terminal Flag tag. Transient transfections with respective plasmids were performed using Polyfect transfection reagent (Qiagen). 48 h after transfection, cells were harvested, lysed and samples further processed for immunoblotting.

### Cell lysis and immunoblotting

Cells were lysed in IP buffer (50 mM Tris-HCl pH 7.5, 150 mM NaCl, 1% NP-40, 5 mM EDTA, 5 mM EGTA, 25 mM NaF, 1 mM orthovanadate, 1 mM PMSF, 10 mg mL^-1^ TPCK and protease cocktail inhibitor from Roche), and cleared by centrifugation at 14,000 rpm for 10 minutes at 4 °C. Total protein concentration was measured using Bradford assay (Bio-Rad). All immunoblotting analysis was performed using 100 μg of total protein. Original uncropped immunoblots are provided in the Source Data file.

### Antibodies

Anti-Total pY (clone 4G10), 1:1000, Millipore # 05-321; anti-Btk (D6T2C), 1:1000, Cell Signaling # 56044; anti-Btk, 1:1000, Thermo Scientific # PA5-27392; anti-Btk (pY551)/Itk (pY511) Clone 24a, 1:1000, BD Biosciences # 558034; anti-Btk (pY223), 1:1000, Cell Signaling #5082; anti-p44/42 MAPK (Erk1/2), 1:1000, Cell Signaling #9102; anti-phospho-p44/42 MAPK (Erk1/2) (Thr202/Tyr204) (E10), 1:1000, Cell Signaling #9106; anti-PLCƴ2, dilution 1:1000, Cell Signaling, #3872; anti-PLCƴ2 (Tyr1217), 1:1000, Cell Signaling #3871; anti-Flag, 1:5000, Sigma # F3165; anti-Penta-his, 1:5000, Qiagen #34660; anti-PLCƴ2 (Tyr753) [EPR5914-3], 1:1000, Abcam #ab133455; anti-Tubulin, 1:2000, Sigma #T9026; anti-Myc-tag Myc.A7 DyLight800, 1:10,000, Thermo Scientific #MA1-21316-D800; anti-mouse IgG IRDye 800CW, 1:10,000, LiCor #926-32210; anti-Rabbit IgG (H+L) IRDye800, 1:10,000, Rockland #611-732-127; anti-Mouse IgG (H+L) Peroxidase AffiniPure, 1:10,000, Jackson ImmunoResearch #115-035-003; anti-Rabbit IgG (H+L) Peroxidase AffiniPure, 1:10,000, Jackson ImmunoResearch #111-035-003. The full information on primary and secondary antibodies that were used for the reported experiments is available in the Reporting Summary.

### Western blot quantification

Quantification using of fluorescent secondary antibodies was done using the Li-Cor Odyssey system. ECL prime detection reagent (RPN2232, GE Healthcare) was used to detect HRP-conjugated antibodies using the C-digit Blot scanner (Li-Cor). Western blot normalization was done using total protein signal or loading control (tubulin).

### Immunoprecipitation

Immunoprecipitation from HEK293 cell lysates was done using 1 mg of total protein adjusted to 1 mL volume with IP buffer. Anti-c-Myc Agarose Affinity Gel (Thermo, A7470) was added to the cell lysates and incubated for 3 h on a rotating wheel at 4 °C. Beads were subsequently washed three times with IP buffer and finally boiled in Laemmli buffer for 5 min at 95 °C before subjected to immunoblotting.

### Mass spectrometry

For confirmation of protein identity and phosphorylation status, recombinant proteins were analyzed on a Xevo G2-S QTOF mass spectrometer (Waters) operated in positive ionization using the ZSpray dual-orthogonal multimode ESI/APCI/ESCi source. Data were processed using MassLynx 4.1 software and MaxEnt1 application for deconvolution.

### B-cell transduction and BCR stimulation

Repebodies containing a C-terminal Flag tag were cloned into the doxycycline-inducible (Tet-ON) lentiviral vector pcW57.1 (Addgene) and co-transfected with envelope and packaging plasmids (pMD2G and pCMVR8.74 respectively, a kind gift from the Trono Lab, EPFL) into HEK293T cells using the CalPhos Mammalian Transfection kit (Clontech). Lentiviruses were concentrated by ultracentrifugation at 30,000xg for 2 h at 16 °C, and added to lymphoma cell lines, followed by a single spinoculation step at 300*g* for 60 min at room temperature. On the next day, cell media was replaced and cells selected using 2 μg mL^−1^ puromycin. Repebodies were induced by the addition of 2 µg mL^−1^ of doxycycline. For BCR stimulation, cells at density 5 × 10^6^ cells mL^−1^ were incubated at 37 °C with 20 µg mL^−1^ anti-human IgM/G F(ab’)2 from goat (Jackson ImmunoResearch, 109-006-129 and 109-006-098) in RPMI-1640 media without calf serum. Cells were then harvested, immediately lysed and samples further processed for immunoblotting.

### Cell viability assays

DLBCL cell lines were treated for 48 h with ibrutinib (concentration range 50 nM to 100 µM) and viability assessed using Cell Titer Glow (Promega). Luminescence was measured in a SpectraMax M5 plate reader (Molecular Devices). DMSO (50 µM) and doxorubicin (10 µM) were used as negative and positive controls, respectively. To assess the effects of repebodies on the viability of DLBCL, transduced cells were treated with 2 µg mL^−1^ of doxycycline to induce expression of repebodies, and cell number verified using a Casy Cell Counter (OLS Omni Life Science). Cell density was maintained at 5 × 10^5^ cells mL^−1^ and regularly diluted when cell density reached 3 × 10^6^ cells mL^−1^. Parental (non-transduced) and non-induced cells were used as control.

### Flow cytometry

Transduced cells were seeded in 6-well cell culture plates, treated with 2 µg mL^−1^ of doxycycline and/or ibrutinib, and stained for the apoptosis marker Annexin V. Briefly, treated cells were washed twice with PBS and resuspended in Annexin V binding buffer containing Cy5-Annexin V (BD, 559934) and 7AAD (BD, 559927) using instructions from the supplier. Cells were gently dissociated and filtered through a 35 µm nylon mesh before data acquisition in a Gallios flow cytometer (BD). A minimum of 10,000 gated events were collected for each sample and data was analyzed using the FlowJo (v10.6) software.

### Molecular dynamics (MD) simulations

System preparation: The Btk SH2-KD structural model used for our MD simulations was built starting from the X-ray structures of the apo-KD of Btk (PDB: 1K2P) and SH2 domain (PDB: 6HTF). The SH2 domain was initially positioned on the top of KD at ~60 Å from it (the distance is measured considering the center of mass of SH2 and N-lobe of KD). The linker sequence was manually added by means of Maestro (Schrödinger Release 2016-1: Maestro, Schrödinger, LLC, New York, 2016) obtaining an extended configuration (Fig. 3a) and subsequently refined through a scaled MD simulation run. Firstly, the protein was parameterized by using the Amber 14SB force field^42^ and immersed in a TIP3P^43^ water box having 12 Å of buffer between the protein and the three edges of the box. The protein was then neutralized by adding an appropriate number of Cl^-^ ions. After minimization, the protein underwent to three NVT simulations steps of 500 ps each to gradually reach the target temperature of 300 K (first step from 0 to 100 K, second step from 100 K to 200 K and last step from 200 K to 300 K). Here, a restraints of 1000 kJ mol^−1^ nm^−2^ was applied to backbone and the velocity-rescaling thermostat^44^ was used. Then, 1 ns of NPT simulation was performed maintaining the restraints and employing the Parrinello-Rahman barostat^45^ to reach the target pressure of 1 bar. Finally, to enhance the sampling of the linker sequence, we performed a 70 ns long Scaled MD simulations^26^, using a *λ* = 0.8, and releasing the restraints for the linker sequence. Electrostatics were treated with the cutoff method for short-range interactions and with the Particle Mesh Ewald method^46^ for the long-range ones (rlist = 1.1 nm, cutoff distance = 1.1 nm, VdW distance = 1.1 nm, PME order = 4). To obtain the optimal configuration of the linker sequence, we performed a cluster analysis on the last 70 ns long trajectory. The centroid of the most populated cluster was then employed as starting point for the next MD simulations. Both the MD simulations and cluster analysis were performed by using BiKi LifeSciences suite^47^.

Scaled MD simulations: We run multiple replicas Scaled MD simulations^26^ to enhance the sampling of Btk kinase and speed up the binding events between SH2 and KD. As starting point, we employed the refined structure of the Btk SH2-KD-linker model (see system preparation section). The equilibrated system was submitted to 40 replicates ~100 ns long Scaled MD simulations using a *λ* = 0.9. Here, restraints were not applied because the high scaling factor enabled adequate sampling without affecting the overall folding of the system. BiKi LifeSciences suite^47^ was used for Scaled MD simulations, using the same settings as in the system preparation section.

Data Analysis: The final aim of our MD studies is to collect all possible Btk SH2-KD bound configurations and to determine which is the most likely bound configuration(s) using both SAXS and mutational studies data. The collected 4 µs-long scaled MD trajectories resulted in a total of 400,000 frames. From this large ensemble, we extracted the unique and non-redundant Btk SH2-KD configurations, using a clustering procedure implemented in BiKi^47^. The resulting 760 structures were submitted to another cluster analysis to probe the preferred 3D structural organization of SH2 domain with respect of KD. Also, we run a CRYSOL analysis^48^ to compute the *χ*^2^ value for each structure, in order to select the SH2-KD complexes with the best fitting with the experimental SAXS curves.

### Development of repebodies

Selection and affinity maturation of human Btk SH2-specific repebody (rF10) were performed through phage display and a modular evolution approach as previously described^28^.

### Small-angle X-ray scattering (SAXS)

SAXS data were collected at the BM29 beamline (ESRF Grenoble, France). All proteins were measured in buffer containing 25 mM Tris pH 7.5, 300 mM NaCl, 1 mM TCEP and 5% glycerol. A robotic sample changer carried out the measurement in the batch mode, while in-line SEC-SAXS was performed using a Superdex S200 Increase 10/300 column (GE Healthcare) with a flow rate of 0.7 mL min^−1^ at room temperature. Acquired data were averaged and subtracted from an appropriate solvent-blank to produce the final curve using the ATSAS Suite, EMBL^49^ and CHROMIXS^50^. Initial data pre-processing and reduction were performed using an automatic pipeline. Final scattering curves were analyzed using PRIMUS for evaluation of molecular dimensions (*R*_g_)^51^ and maximum particle dimension (*D*_max_) using GNOM^52^. Moreover, the Porod volume was computed using the Porod invariant^53^, and the molecular mass estimated using SAXSMoW 2.0^54^, Bayesian inference approach^55^ and Volume-of-correlation^56^. Ab initio models were computed with DAMMIF^57^. SREFLEX^50^ was employed to improve the agreement of flexible multidomain models to the experimental data. Finally, the flexibility of multidomain complexes was assessed with Ensemble Optimization Method 2.0^27^. Fitting of models to experimental data was assessed using CRYSOL^48^ molecular and superimpositions performed with the SASpy^58^. Data collection and structure determination statistics are described in Supplementary Tables 2 and 4.

### Structure determination

Recombinant Btk SH2 and rF10 proteins were mixed at 1:1 ratio and the complex purified with a Superdex 75 column 16/600 (GE Healthcare) in buffer containing 25 mM Tris pH 7.5, 300 mM NaCl, 1 mM TCEP. The purified complex was concentrated to ~25 mg mL^−1^ and crystallized at 18 °C using the hanging-drop vapor-diffusion method by mixing 1:1 with a solution containing 1 M Tris pH 8.5, 300 mM sodium fluoride, 300 mM sodium bromide, 300 mM sodium iodide, 25% MPD; 25% PEG 1000; 25% PEG 3350. 20% glycerol was used as a cryoprotectant. X-ray diffraction data was collected at the SLS Beamline X06DA in the Swiss Lightsource (SLS, Villigen, Switzerland) at a wavelength of 1 Å and temperature of 100 K. Data collection and structure determination statistics are described in Supplementary Table 3. Diffraction data was processed and scaled with the XDS package. The structure was solved by molecular replacement employing models derived from a previously reported repebody (PDB 5B4P) and Btk SH2 (PDB 2GE9) excluding loop regions. Molecular replacement, manual model building, B-factor refinement, solvent addition, energy-minimization and refinement of structures were conducted iteratively using Phaser and Coot (Phenix version 1.13). Molecular graphics were generated using PyMOL (DeLano Scientific).

### Isothermal titration calorimetry (ITC)

Proteins were extensively dialyzed in buffer containing 20 mM Hepes pH 7.5 and 150 mM NaCl, briefly degassed, and concentration determined by measuring UV absorbance at 280 nm. ITC measurements were performed on a MicroCal PEAQ-ITC (Malvern) instrument. The repebody (100 µM) was titrated into SH2 domains (10 µM) at room temperature in 16 steps with 0.49 μL for the first and 2.49 μL for the other steps. Thermodynamic parameters were obtained using the MicroCal software.

### Fluorescent Polarization (FP) binding assays

Btk SH2 WT and mutants were incubated at several concentrations with 1 µM of FITC-labeled peptide (ADNDpYIIPLPD) in Tris 40 mM pH 8, 150 mM NaCl and 1 mM DTT. Competitive FP assay was performed using 25 µM of Btk SH2 and 1 µM of peptide incubated with repebody in a range of 200 µM–20 nM. FP signal was measured using a SpectraMax M5 plate reader (Molecular Devices) with excitation at 485 nm and emission at 530 nm in a 96 well black-plate (Greiner).

### Multi-angle light scattering analysis (SEC-MALS)

Multi-angle light scattering was used to probe for oligomerization states. All measurements were performed at room temperature using a Dawn Heleos multi-angle light scattering detector (Wyatt Technologies) coupled to an SEC column. 80 μL (0.5 mg mL^−1^) of purified recombinant protein was injected into a Superdex 75 HR10/30 column (GE Healthcare) in buffer containing 25 mM Tris-HCl pH 7.5, 150 mM NaCl, 1 mM TCEP, and eluted at a flow rate of 0.5 mL min^−1^. Absolute molecular weight and homogeneity were determined using ASTRA version 5.3 (Wyatt Technologies).

### Circular dichroism (CD)

Far-UV spectra (190–300 nm) of recombinant Btk SH2 WT and mutants were carried out in buffer containing 10 mM Na-phosphate buffer pH 7.2 and 100 mM NaF using a 0.1 cm quartz cell and CD Spectrometer Chirascan V100 (AppliedPhotophysics). Data was acquired at a step size of 1 nm and bandwidth of 1 nm. 3 scan records for each protein were subtracted from the background (buffer only) and averaged to generate the data reported in units of mean molar ellipticity per residue. Melting curve analysis was performed by measuring proteins at the wavelength corresponding to the peak for the predominantly ß-sheet SH2 domain (218 nm) in a temperature range from 20 to 94 °C, ramp-rate 1 °C per minute.

### In vitro Kinase assay

2 µM of repebodies were pre-incubated with 1 µM of recombinant Btk proteins in buffer Tris 25 mM pH 7.5, 150 mM NaCl, 5% glycerol, 20 mM MgCl_2_, 1 mM DTT in the presence of 50 μM ATP, 7 μCi γ-^32^P-ATP, and PLCγ2 peptide carrying an N-terminal biotin (biotin-ERDINSLYDVSR-amide). Peptide concentrations ranged from 100 μM to 30 μM. Reactions were carried out on a final volume of 20 µL at room temperature for 20 min and terminated using 10 μL 7.5 M guanidinhydrochlorid. Samples were spotted onto a SAM2 Biotin Capture membrane (Promega) and further treated according to the instructions of the manufacturer.

### Mapping of Btk autophosphorylation sites

Sample preparation: Recombinant autophosphorylated Btk SH2-KD was separated by SDS-PAGE and stained with Coomassie. Bands of interest were excised, in-gel digested in reduced in 10 mM DTE, 50 mM AB, and then alkylated in 55 mM iodoacetamide, 50 mM AB. After a washing step, gel extracts were digested with MS Grade Trypsin over-night. Resulting peptides were finally extracted using a high organic containing solvent and dried by vacuum centrifugation prior to LC-MS2 measurements or phosphopeptides enrichment.

Next, 90% of the extracted peptide was used for phosphopeptides enrichment step while the remaining 10% was used for sample identification. Titanium dioxide affinity principle was used for enrichment using home-made titania tips (based on Thingholm and Larsen 2009). Dried samples were resuspended 0.75% TFA, 60% acetonitrile, 300 mg mL^−1^ lactic acid, loaded on tips, and eluted in 0.5% ammonium hydroxide and 5% piperidine. Samples were acidified and dried down prior to LC-MS2 measurements.

MS analysis: For the MS detection of phosphopeptides, dried samples were resuspended in 0.1% TFA and separated by C18 Reverse Phase nano UPLC using a Dionex Ultimate 3000 RSLC system (Thermo Fischer) connected to an Orbitrap Elite Mass Spectrometer (Thermo Fischer). Samples were first trapped on a home-made capillary C18 pre-column and then separated on a C18 capillary column (Nikkyo Technos Co; Magic AQ C18; 3 µm - 100 Å; 15 cm × 75 µm ID). Data-dependent mode was used for MS acquisitions were the 20 most intense parent ions were selected for subsequent fragmentation by CID. A potential phosphopeptides *m*/*z* inclusion list was also generated and used to maximize detection chances.

### Quantification and statistical analysis

All data reported were analyzed using Prism 7 (GraphPad) using software-defined fitting models and unpaired *t*-test statistical test. Calculated *P*-values are indicated as non-significant (ns), *P* ≤ 0.05 (*), *P* ≤ 0.01 (**), *P* ≤ 0.001 (***) and *P* ≤ 0.0001 (****).

### Reporting Summary

Further information on research design is available in the Nature Research Reporting Summary linked to this article.

## Supplementary information

Supplementary Information

Peer Review File

Reporting Summary

Description of Additional Supplementary Files

Supplementary Movie 1

## Data Availability

The data that support the findings of this study are available from the corresponding author upon reasonable request. The X-ray structure of the rF10-SH2 complex was deposited at Protein Data Bank, PDB 6HTF [https://www.rcsb.org/structure/6HTF]. Full SAXS curves and analyzed data for wild-type Btk proteins were deposited at SASBDB [https://www.sasbdb.org/data] with accession numbers SASDF53, SASDF63, SASDF73, and SASDF83.

## Source data

Source Data
