# Supplementary material for: Btk SH2-kinase interface is critical for allosteric kinase activation and its targeting inhibits B-cell neoplasms
Source: Nat Commun. 2020 May 8;11:2319. doi: 10.1038/s41467-020-16128-5 (PMC7210950; doi:10.1038/s41467-020-16128-5)
Supplement: Supplementary file 1 — Supplementary Information [file 41467_2020_16128_MOESM1_ESM.pdf]

SUPPLEMENTARY INFORMATION

Btk SH2-kinase interface is critical for allosteric kinase activation and its targeting inhibits B-cell neoplasms

Duarte et al.

Contents

Supplementary Figures 1-7

Supplementary Tables 1-5

SUPPLEMENTARY FIGURES 1-7

Supplementary Figure 1

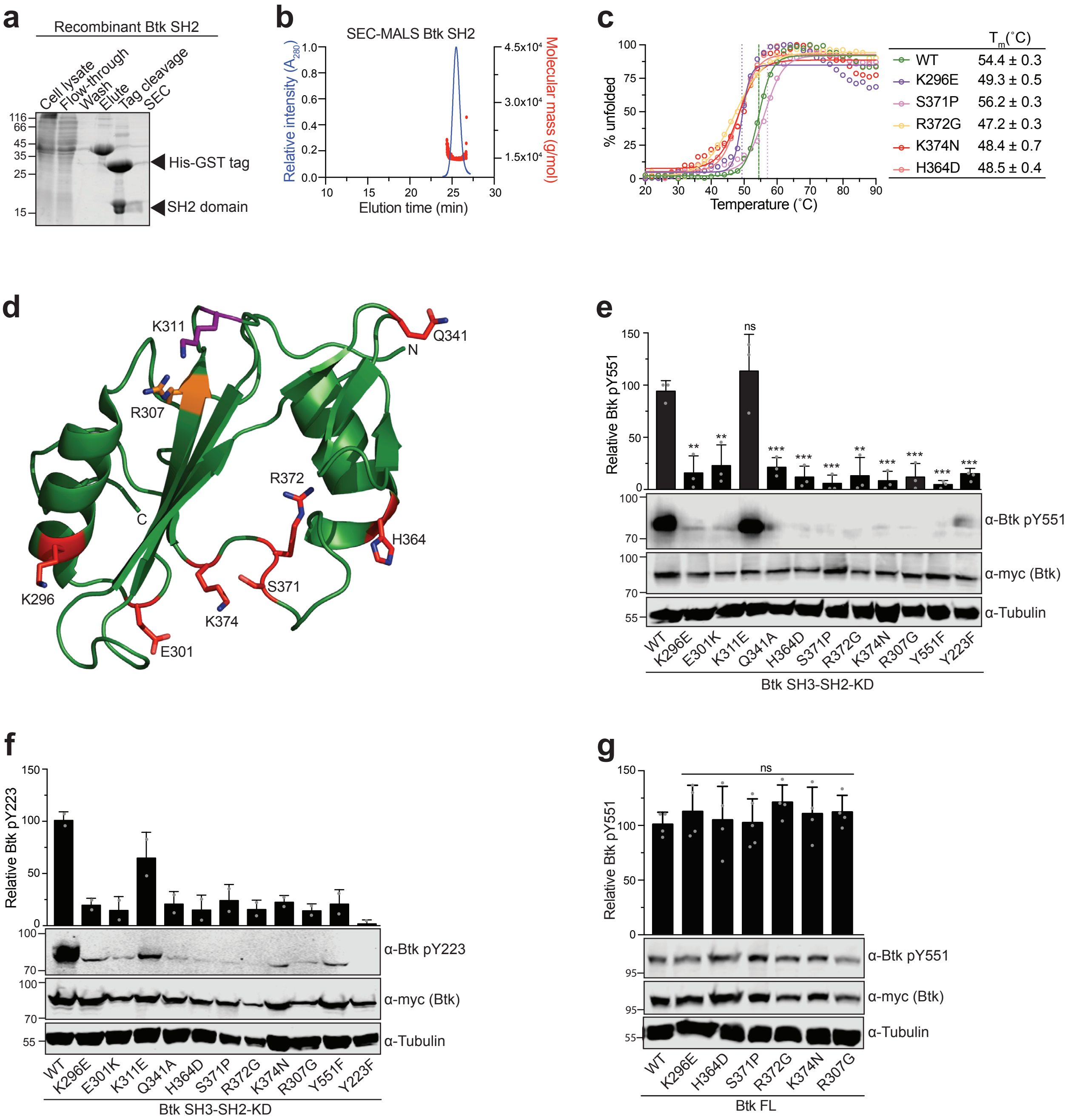

Supplementary Figure 1, related to Figure 1. Effect of XLA mutations *in vitro* and in HEK cells.
(**a**) Representative SDS-PAGE analysis of purification steps for recombinant Btk SH2 wild-type from *E.coli*. TEV cleavage was used to removal of 6xHis-GST tag used for purification. All Btk SH2 mutants were purified using an identical protocol.
(**b**) Representative SEC-MALS analysis of purified Btk SH2 domain wild-type (monomer = 13.2 kDa). All proteins were analyzed by SEC-MALS and found in the homogenous state in solution (data not shown).
(**c**)Thermal shift assay (TSA) of recombinant Btk SH2 domains. Melting temperature (T_m) for wild-type Btk was calculated from two independent measurements.
(**d**) Mapping of a subset of XLA-patient mutations (red sticks) onto the human Btk SH2 structure (PDB 2GE9). The residue R307 (orange sticks) is part of the pY-binding motif (FIVRD). The residue K311 is a non-XLA control mutation facing the opposite surface of the SH2 domain. N- and C-terminal are indicated as N and C, respectively.
(**e,f,g**) HEK293 cells were transiently transfected with indicated Btk constructs containing an N-terminal 6xMyc tag. Immunoblotting of total cell lysates was performed to assess Btk phosphorylation on sites Y551 and pY223, and relative phosphorylation normalized to total Btk (Myc-Btk) expression. Tubulin was used as loading control. Data shown in (e) and (g) are the mean ± SD of three biological replicates (n=3), while data shown in (f) is the mean ± SD of two technical replicates (n=2). P-values were calculated against the wild-type (WT) using an unpaired *t*-test. **P ≤ 0.01, ***P ≤ 0.001, and non-significant (ns).
Source data are provided as a Source Data file.

Supplementary Figure 2

a

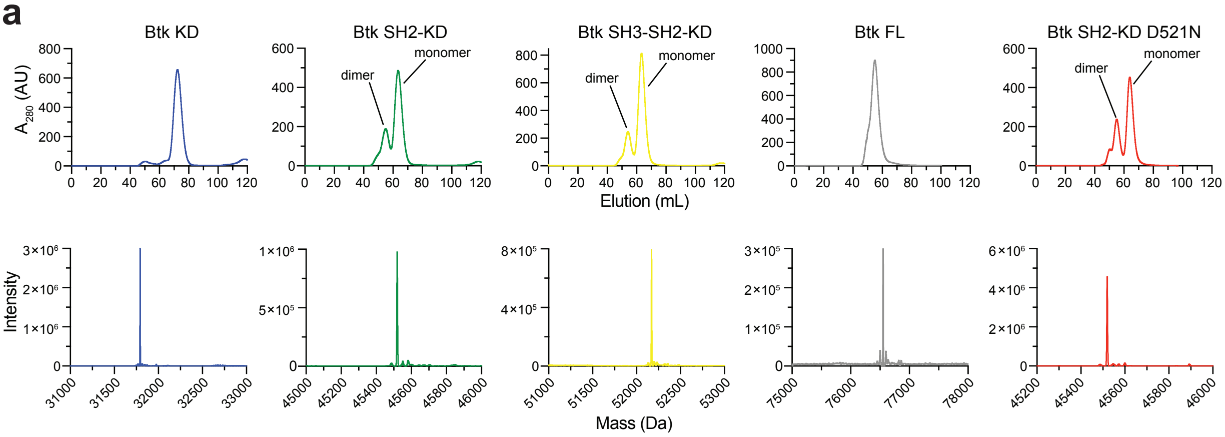

Mass spectrometry analysis

	Btk KD	Btk SH2-KD (monomer)	Btk SH3-SH2-KD (monomer)	Btk FL	Btk SH2-KD D521N (monomer)
Obtained (Da)	31794.09	45520.35	52171.90	76553.45	45520.19
Theoretical (Da)	31793.52	45519.93	52171.42	76553.51	45518.95

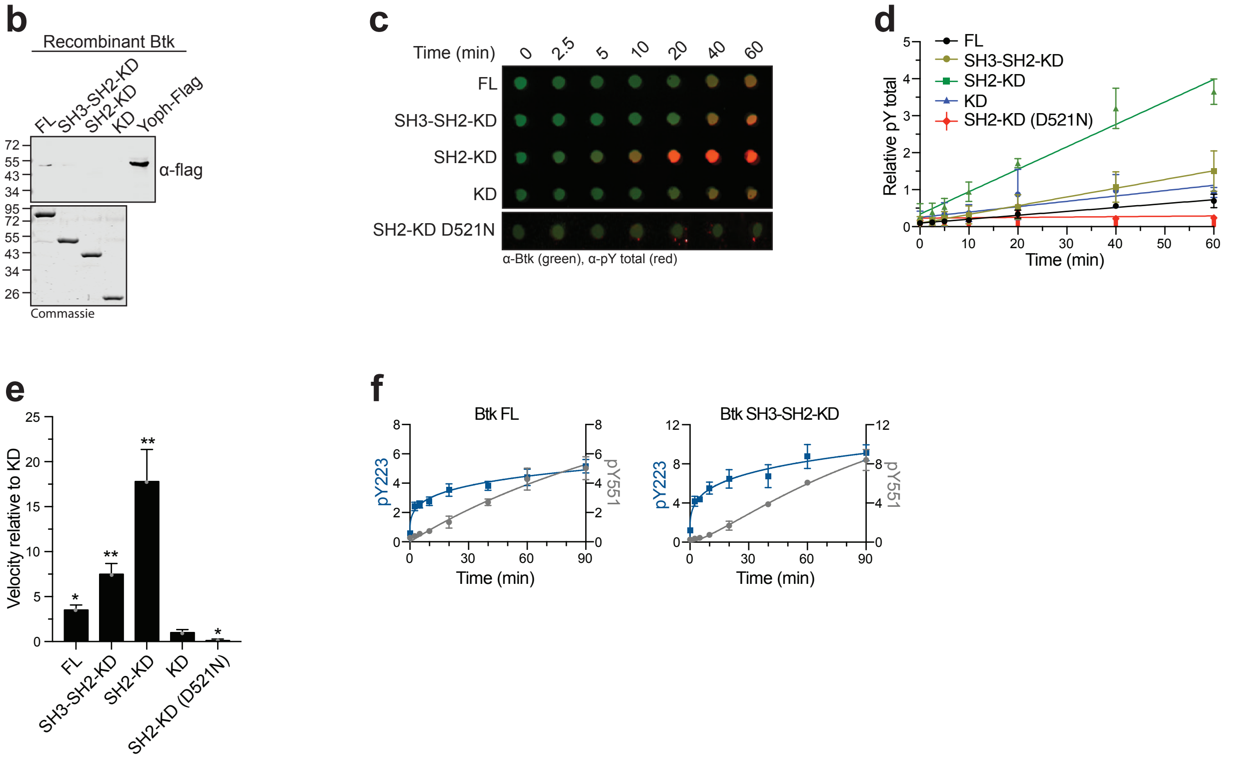

Supplementary Figure 2, related to Figure 2. Purification of recombinant Btk and autophosphorylation *in vitro*.
(**a**) SEC of recombinant Btk expressed and purified from Sf9 cells (top). Only monomeric peaks were used for the described assays. All samples were subjected to MS analysis for confirmation of protein identity and unphosphorylated state (> 95% for all samples, bottom). The table summarizes the theoretical and obtained molecular weight for the indicated proteins.
(**b**) Representative immunoblot to confirm the absence of recombinant Yoph-flag phosphatase from proteins purified in Sf9 cells (top) and corresponding SDS-PAGE of recombinant untagged Btk proteins (bottom).
(**c,d,e**) Btk autophosphorylation *in vitro* assay performed as described in methods. The levels of total phosphotyrosine and total Btk were assessed using immunoblot in a dot-blot apparatus, relative autophosphorylation kinetics plotted overtime and normalized to total Btk protein, and relative autophosphorylation velocities calcurated from the linear fitting. Data are the mean ± SD of two independent experiments (n=3). P-values relative to Btk KD were calculated using unpaired *t*-test. *P ≤ 0.05 and **P ≤ 0.01.
(**f**) Autophosphorylation kinetics of Y223/Y551 *in vitro* assessed as described above. Data shown are the mean ± SD of two independent experiments done in duplicates (n=2).
Source data are provided as a Source Data file.

Supplementary Figure 3

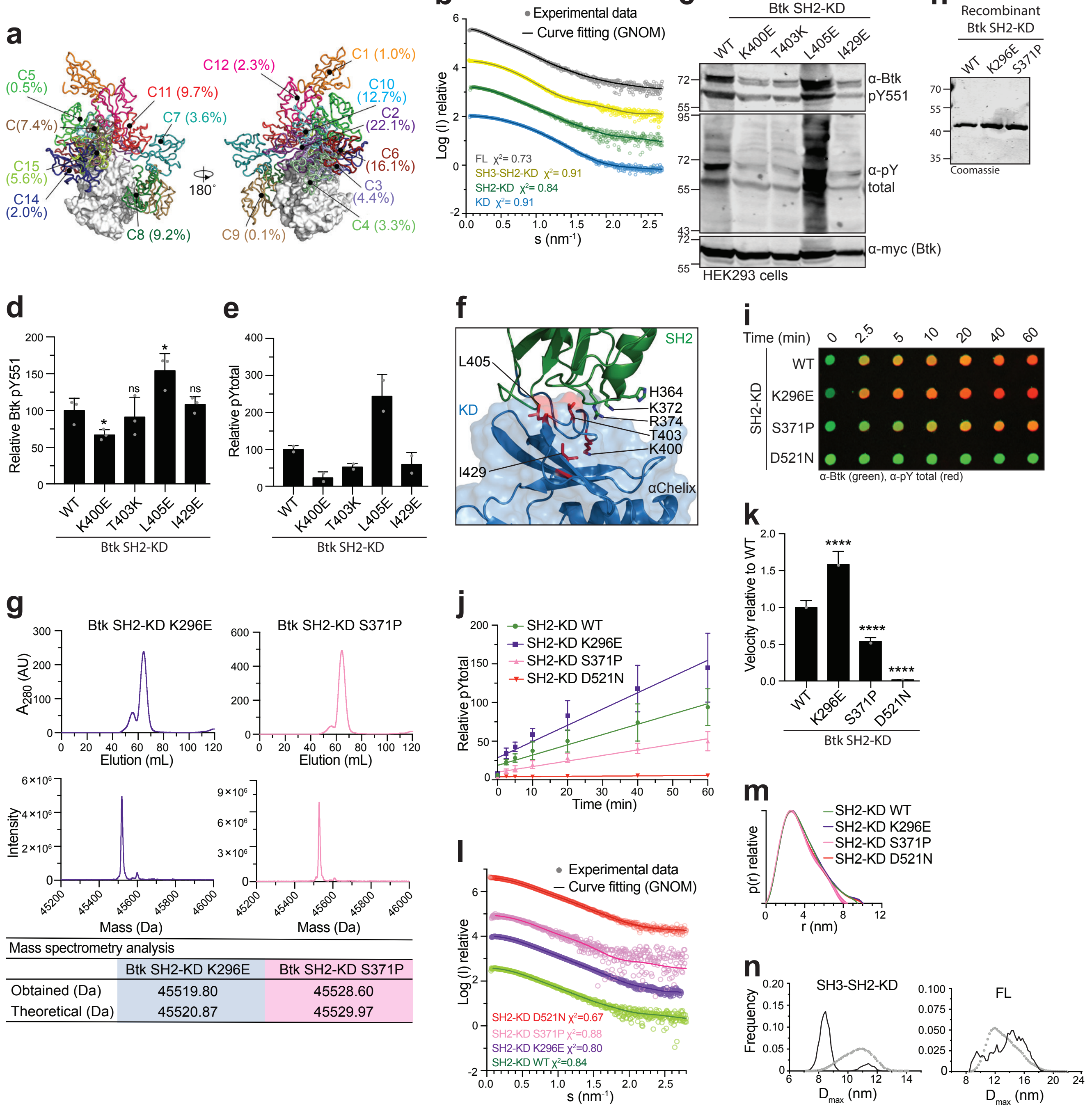

Mass spectrometry analysis

	Btk SH2-KD K296E	Btk SH2-KD S371P
Obtained (Da)	45519.80	45528.60
Theoretical (Da)	45520.87	45529.97

Supplementary Figure 3, related to Figure 3 and 4. MD and SAXS analysis of Btk wild-type and mutants.
(**a**) MD simulation for the SH2-KD complex. Obtained clusters of the SH2 positions (several colors) relative to the KD (white).
(**b**) Experimental SAXS data of recombinant wild-type Btk proteins. The indicated $\chi2$ represents the GNOM fitting (line) against the experimental data (dots) for each construct. See Supplementary Table 2 for details. Raw data is available at SASDB.
(c,d,e) HEK293 cells were transiently transfected with indicated Btk constructs containing an N-terminal 6xmyc tag. Immunoblotting of total cell lysates was performed to assess Btk phosphorylation on Y551 and pY total, and relative phosphorylation normalized to total Btk (Myc-Btk) expression. Tubulin was used as loading control. Data shown in (d) and (e) are the mean ± SD of three (n=3) and two technical (n=2) replicates, respectively. P-values relative to Btk WT were calculated using an unpaired *t*-test.
(f) Residues mutated are shown as red sticks in a representative Btk SH2-KD structure obtained in the MD simulation (C15).
(**g**) SEC of recombinant mutant Btk purified from Sf9 cells (top). All samples were subjected to MS analysis for confirmation of protein identity and unphosphorylated state (>95% for all samples, bottom).
(**h**) Representative SDS-PAGE analysis of recombinant untagged Btk SH2-KD mutant proteins purified from Sf9 cells.
(**i,j,k**) *In vitro* autophosphorylation of Btk SH2-KD mutants performed as described in methods. The levels of total phosphotyrosine and total Btk were assessed using immunoblot in a dot-blot apparatus, relative autophosphorylation kinetics plotted overtime and normalized to total Btk protein, and relative autophosphorylation velocities obtained from linear fit. Data are the mean ± SD of twoindependent experiments (n=6). P-values relative to Btk wild-type (WT) were calculated using unpaired *t*-test. *P ≤ 0.05, **P ≤ 0.01, ***P ≤ 0.001, ****P ≤ 0.0001, and non-significant (ns).
(**i,m**) Experimental SEC-SAXS data and D_{max} of mutant Btk proteins as indicated in (b). See Supplementary Table 2 for details.
(**n**) Flexibility analysis (EOM 2.0) of Btk WT showing the D_{max} of selected conformers (lines) from a representative pool of theoretical conformations (dot line).
Source data are provided as a Source Data file.

Supplementary Figure 4

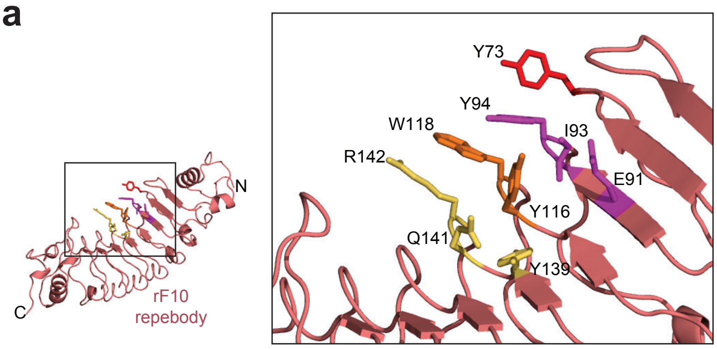

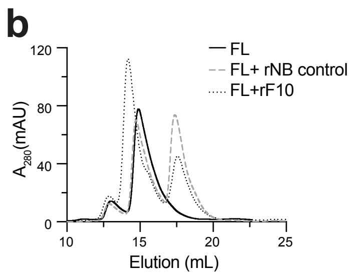

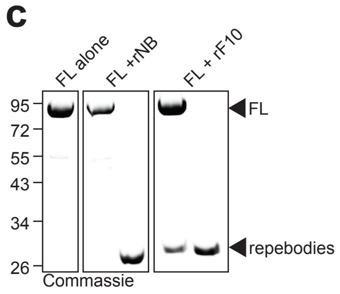

Diversified residues in the Btk SH2 repebody (rF10)

	LRRV1	LRRV2			LRRV3		LRRV4			K_d (nM)
Position	73	91	93	94	116	118	139	141	142	
rNB (template)	N	I	T	G	V	E	N	A	H	N.B.
rF10	Y	E	I	Y	Y	W	Y	Q	R	15.5±1.3

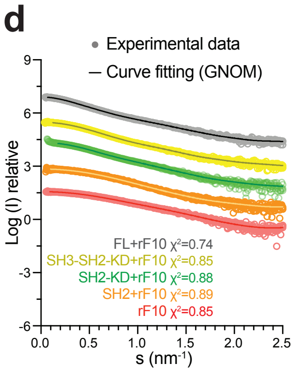

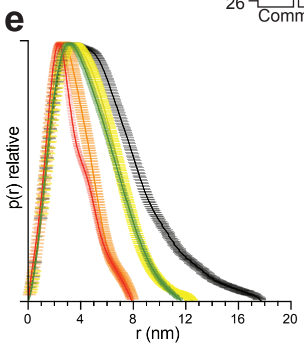

Structural parameters obtained from SAXS

	rF10	rF10+SH2	SH2-KD+rF10	SH3-SH2-KD+rF10	FL+rF10
R_g(nm)	2.42±0.2	2.6±0.14	3.48±0.17	3.55±0.26	4.48±0.38
D_{max}(nm)	7.9±0.8	8.2±0.8	11.5±1.2	12.5±1.2	16.9±1.6

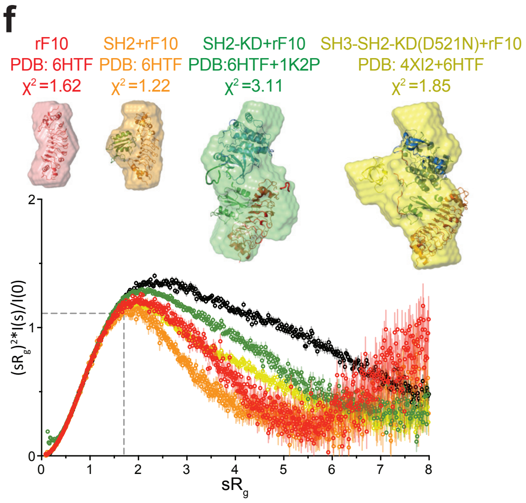

Supplementary Figure 4, related to Figure 5. rF10 repebody development and SAXS analysis of Btk-rF10 complexes.
(**a**) The rF10 repebody (cartoon representation, salmon) was developed by randomizing variable sites within leucine-rich repeats (LRRV) using phage display and modular evolution approach. The residues from the LRRV1 (red), LRRV2 (magenta), LRRV3 (orange) and LRRV4 (yellow) mediating the binding to Btk SH2 domain are indicated as sticks. The table shows the amino acid sequence and binding affinity to the human Btk SH2 domain. Non-binding (N.B.).
(**b**) Size-exclusion chromatogram (SEC) analysis of full-length Btk alone and mixed with rF10 or rNB control. The FL+rF10 forms an stable 1:1 complex which shifts to the left.
(**c**) Peaks isolated from the SEC analysis shown in (b) resolved by SDS-PAGE and stained with Coomassie.
(**d,e**) Experimental SEC-SAXS data and D_{max} for rF10 alone and rF10-Btk complexes. The indicated χ2 represents the GNOM fitting (line) against the experimental data (dots) for each respective sample. The table summarizes the particle dimensions (R_g and D_{max}) and the ± error for the indicated constructs. See Supplementary Table 4 for details.
(**f**) Dimensionless Kratky plot of rF10 alone and rF10-Btk complexes. *Ab initio* reconstructions obtained from SAXS (surface representation) were superimposed to the indicated crystal/MD structures. For the rF10-SH2-KD and rF10-SH3-SH2-KD complexes, rigid body modeling using SASREF was applied to obtain the final models displayed.
Source data are provided as a Source Data file.

Supplementary Figure 5

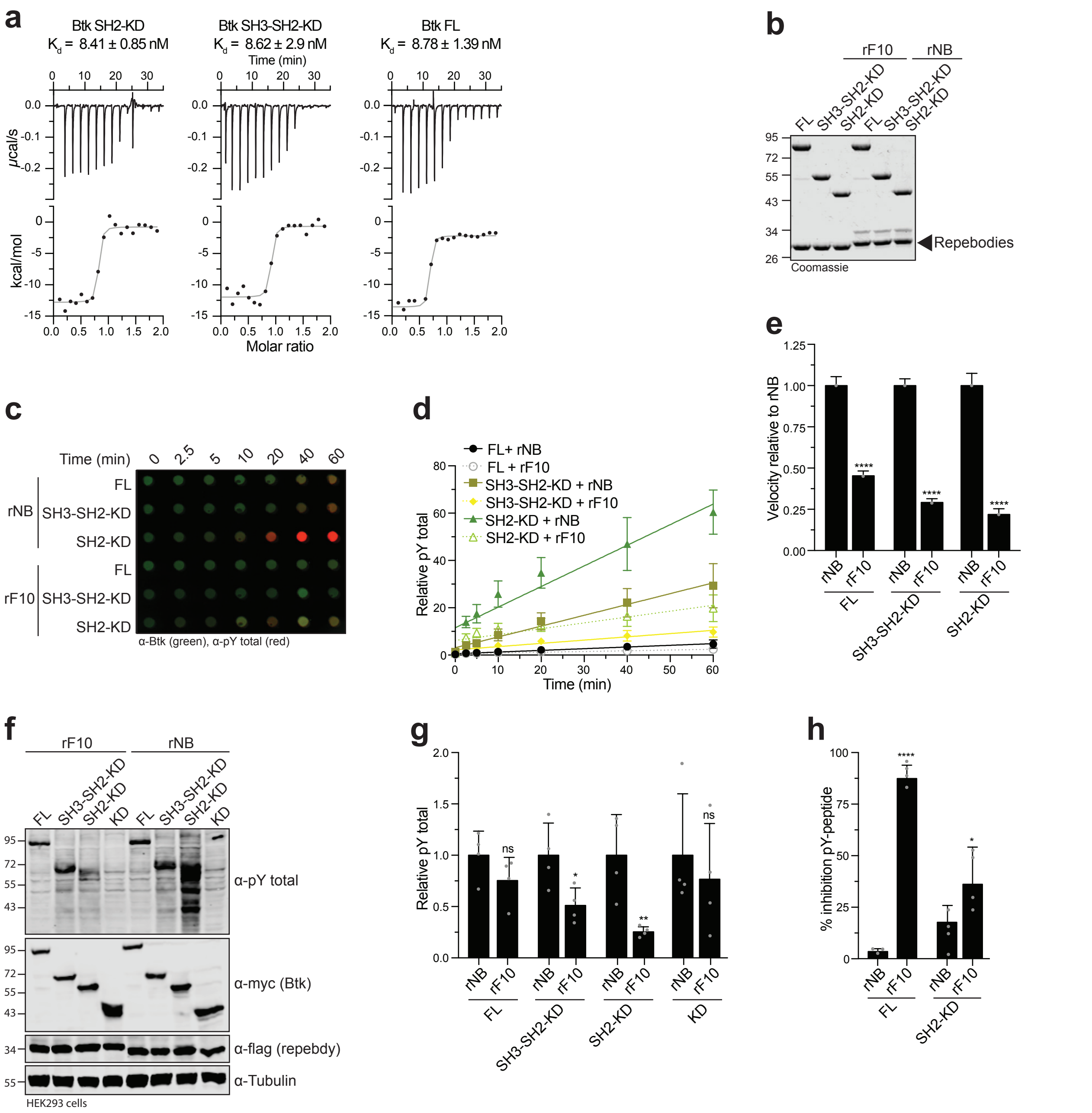

Supplementary Figure 5, related to Figure 6. Functional characterization of the rF10 repebody.

(**a**) ITC measurement of rF10 repebody to different Btk constructs containing the SH2 domain (SH2-KD, SH3-SH2-KD and FL proteins). Top panels show the raw signal from a representative measurement, and bottom panels show the integrated calorimetric data of the area of each peak. The continuous line indicates the best fit to the experimental data assuming a 1:1 binding model. The K_d (± SD) value was calculated from two independent measurements.

(**b**) Representative SDS-PAGE analysis of recombinant Btk proteins mixed with rF10 and rNB control repebodies and used for autophosphorylation inhibition *in vitro*.

(**c,d,e**) *In vitro* autophosphorylation assay for Btk proteins in the presence of indicated repebodies was performed as described in methods. The levels of total phosphotyrosine and total Btk were assessed using immunoblot in a dot-blot apparatus. Relative autophosphorylation kinetics in the presence of rF10 (dashed lines) or rNB (continuous lines) repebodies plotted overtime and normalized to total Btk protein, and relative autophosphorylation velocities relative to each control repebody. Data are the mean ± SD of three independent experiments (n=4). P-values relative to each control rNB repebody were calculated using an unpaired *t*-test.

(**f,g**) HEK293 cells were transiently co-transfected with indicated Btk constructs and repebodies. Immunoblot was used to assess total phosphotyrosine phosphorylation. Quantification of total phosphotyrosine normalized to total Btk (Myc-Btk) expression level and relative to control repebody. Data shown are the mean ± SD of three biological replicates (n=4), and P-values were calculated relative to each rNB control using an unpaired *t*-test.

(**h**) Btk kinase activity against a PLCγ2 peptide (ERDINSL$_{753}$YDVSR) in the presence of rF10 or control repebody. Reported inhibition (% of inhibition of peptide phosphorylation) from two independent experiments done in duplicates (n=4). P-values were calculated relative to each rNB control using an unpaired *t*-test. *P ≤ 0.05, **P ≤ 0.01, ***P ≤ 0.001 and non-significant (ns).

Source data are provided as a Source Data file.

Supplementary Figure 6

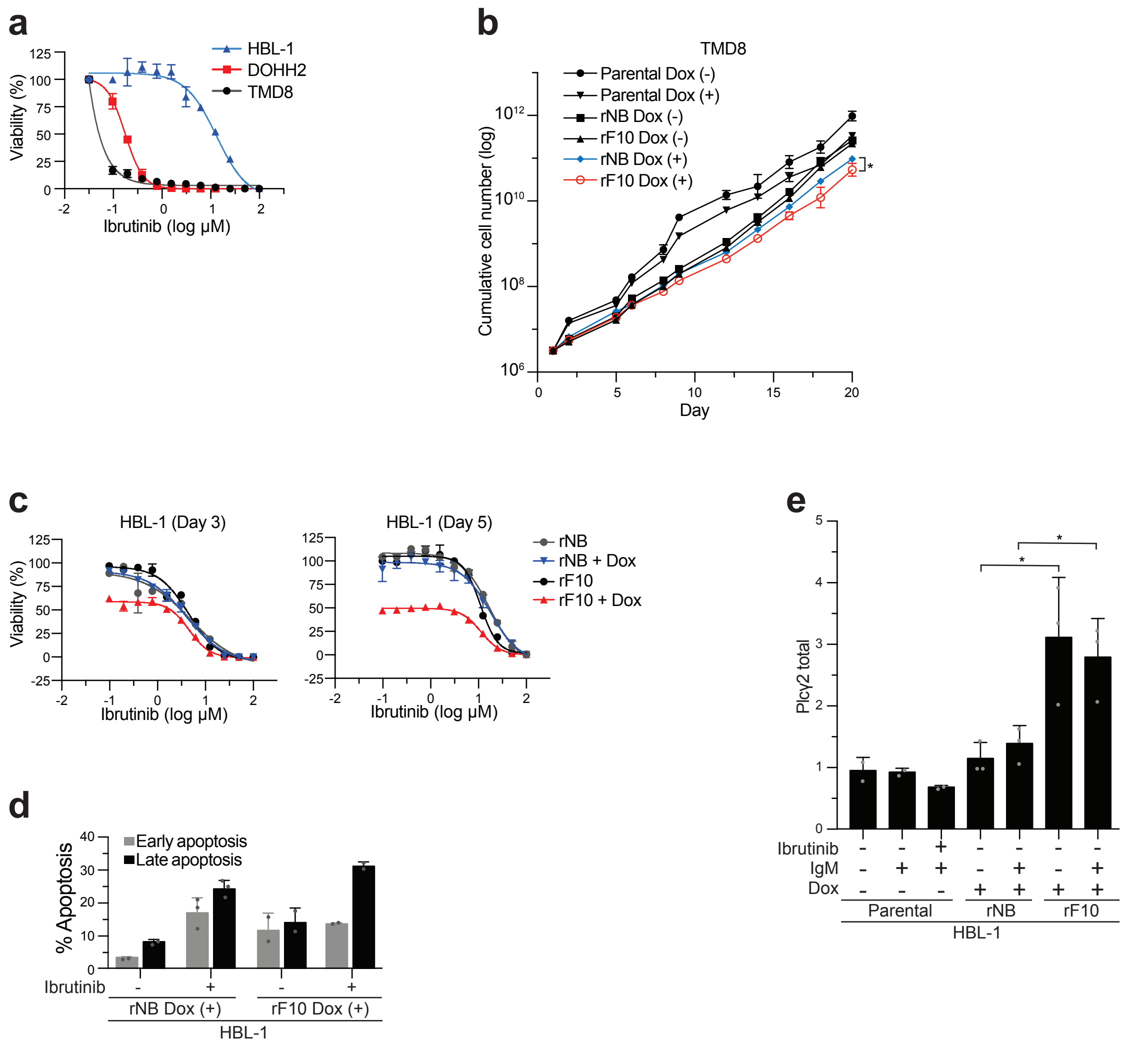

Supplementary Figure 6, related to Figure 7. Effect of rF10 in DLBCL cell lines.
(**a**) Dose-response for ibrutinib in human DLBCL cell lines. Data points represent the mean ± SD of a representative experiment done in duplicates (n=2). Cellular IC50 was obtained by non-linear regression curve fit analysis.
(**b**) TMD8 cell line was transduced with a doxycycline-inducible system for expression of repebodies, and cumulative cell number monitored upon treatment with 2 µg mL^{-1} of doxycycline (n=3). Parental cells are non-transduced cells.
(c) Viability of HBL-1 cells inducibly expressing rF10 and rNB control (+Dox) or non induced (-Dox) in the presence of ibrutinib was measured after 3 and 5 days using Cell Titer-Glo reagent. Data points represent the mean ± SD of a representative experiment done in duplicate (n=2). Cellular IC50 was obtained by non-linear regression curve fit analysis.
(d) HBL-1 inducibly expressing rF10 or rNB control for 5 days in combination with ibrutinib (10µM for 48 hours) were stained with 7AAD and Annexin V to analyze apoptosis by FACS. The quantification of early (7AAD-/Annexin V+) and late (7AAD+/Annexin V+) apoptotic cells were obtained from two replicates (n=2).
(**e**) Quantification of total PLCγ2 level from HBL-1 inducibly expressing repebodies (flag-tagged) for 48 hours. BCR stimulation and ibrutinib treatment were performed as described in methods. Data shown are the mean ± SD from two biological replicates (n=3), and P-values were calculated using unpaired *t*-test. *P ≤ 0.05 (n=3).
Source data are provided as a Source Data file.

Supplementary Figure 7

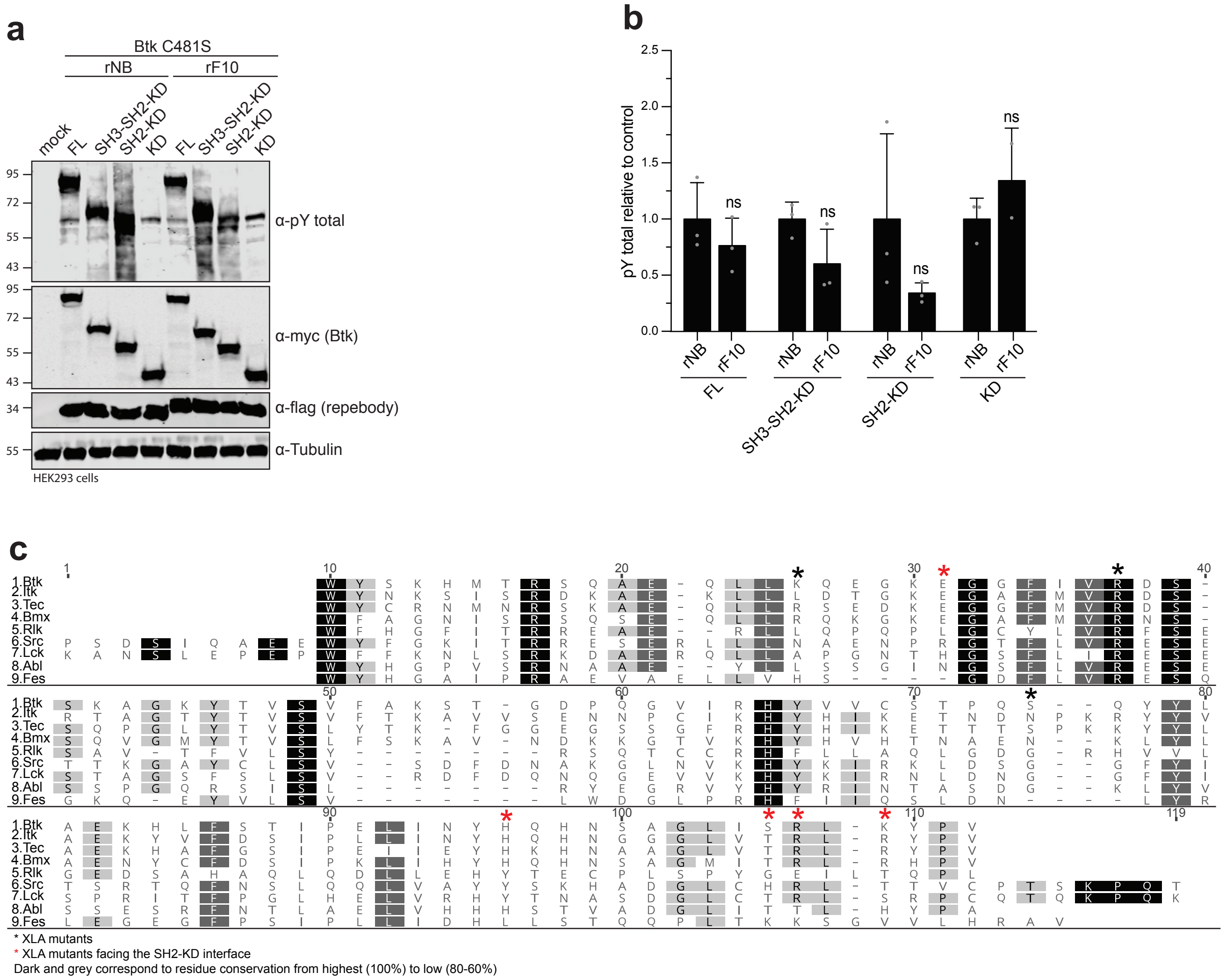

Supplementary Figure 7, related to Figure 8 and Discussion. Targeting the Btk SH2-KD interface decreases activation of therapy-resistant Btk with mutation on C481.

(**a,b**) HEK293 cells were transiently co-transfected with indicated Btk-C481S constructs and repebodies. Immunoblot was used to assess total phosphotyrosine phosphorylation. Quantification of total phosphotyrosine normalized to total Btk (myc-Btk) expression level and relative to control repebody. Data shown are the mean ± SD of two biological replicates (n=3). (**c**) Sequence alignment of SH2 domains from human Btk and related kinases. Black squares represent residues highly conserved and grey squares show residues relatively conserved. Black asterisk symbol (*) indicates residues mutated in XLA patients, while red asterisks are residues mutated in XLA patients and located in the predicted SH2-KD interface.

Source data are provided as a Source Data file.

SUPPLEMENTARY TABLES 1-5

Supplementary Table 1. *In vitro* autophosphorylation sites on the Btk SH2-KD protein.

Site	Peptide sequence	Location	Peptide count
Y279	SDSIEMYEWYSK	SH2	56
Y282	SDSIEMYEWYSK	SH2	56
Y315	AGKYTVSVFAK	SH2	60
Y334	STGDPQGVIRHYVVCSTPQSQYYLAEK	SH2	46
Y344	STGDPQGVIRHYVVCSTPQSQYYLAEK	SH2	46
Y345	STGDPQGVIRHYVVCSTPQSQYYLAEK	SH2	46
Y361	HLFSTIPELINYHQHNSAGLISRLK	SH2	4
Y375	YPVSQQNK	SH2-KD linker	3
Y392	NAPSTAGLGYGSWEIDPK	SH2-KD linker	10
Y425	WRGQYDVAIK	KD	10
Y461	LVQLYGVCTK	KD	2
Y511	DVCEAMEYLESK	KD	4
Y545	VSDFGLSRYVLDDEYTSSVGSK	KD	37
Y551	VSDFGLSRYVLDDEYTSSVGSK	KD	37
Y571	FPVRWSPPEVLMYSK	KD	15
Y627	VYTIMYSCWHEK	KD	6
Y631	VYTIMYSCWHEK	KD	6
	Total peptide count		444

Supplementary Table 2. SAXS parameters of human Btk wild-type and mutants.

**Data collection parameters**	
nstrument	MD29 beamline, ESRF Grenoble - France
Wavelength (Å)	0.9919
q-range (nm$_{-1}$)	0.03563 - 5
Exposure time (sec)	5 (10 frames x 0.5 sec)
Temperature (K)	290

**Samples**	**KD**	**SH2-KD**	**SH3-SH2-KD**	**Full-length**	**SH2-KD K296E**	**SH2-KD S371P**	**SH2-KD D521N**
Measurement mode	batch	SEC-SAXS	batch	batch	SEC-SAXS	SEC-SAXS	batch
Concentration range (mg.ml$_{-1}$)	0.9 – 4.2	100µ at 20.4	0.9 – 9.2	0.4 – 5.5	100µl at 30	100µl at 10.8	0.6 – 1.9
SASBDB identifier	SASDF53	SASDF63	SASDF73	SASDF83	N/A	N/A	N/A
**Structural parameters**							
R_g (nm) from Guinier	2.09 ± 0.02	2.832 ± 0.3	2.62 ± 0.15	4.04 ± 0.03	2.92 ± -0.04	2.64 ± 0.23	2.83 ± 0.07
I(0)* (cm$_{-1}$) from Guinier	24.86 ± 0.036	85.5 ± 0.12	44.28 ± 0.056	60.24 ± 0.16	171.59 ± 0.15	8.66 ± 0.075	20.71 ± 0.044
R_g (nm) from P(r)	2.096 ± 0.0003	2.88 ± 0.0005	2.62 ± 0.0006	4.34 ± 0.0014	2.95 ± 0.0003	2.70 ± 0.03	2.9 ± 0.05
D_{max} (nm)	6.75 ± 0.67	9.6 ± 0.95	8.3 ± 0.83	15.5 ± 1.2	10 ± 1.0	8.5 ± 0.86	10.3 ± 1.2
Porod volume (nm$_3$)	52.4	65.9	72.44	114.07	66.47	62.53	60.82
Dry volume calculated from sequence (nm$_3$)**	38.468	55.077	63.126	92.784	55.059	55.89	55.076
**Molecular mass determination (kDa)**							
From Porod volume (V_p/~1.6)	24.9	47.6	40.9	76.1	48.5	39.1	42.7
From SAXS MoW2***	28.9	49.7	35.7	65.2	50.9	44.6	46.4
Bayesian inference	28.9	46.6	41.9	67.1	46.6	42.8	40.2
From I(0) using V_c invariant	28.2	43.5	43.4	65.6	43.7	40.2	42.7
Calculated from sequence****	31.8	45.5	52.2	76.5	45.5	45.5	45.5
Number of residues	274	396	452	664	396	396	396

**Software list**	
Primary data reduction	Automated pipeline at beamline
Data processing	PRIMUS (ATSAS v.2.8.0)
Ab initio analysis	DAMMIN and GASBOR
Fitting	CRYSOL
Model refinement	SREFLEX
Flexibility analysis	EOM 2.0
Model superimpositions	SASpy plugin for Pymol
3D graphics images	Pymol (v.1.8.2.1)

*I(0) values shown in SEC-SAXS measurements vary depending on protein concentration at the analyzed peak, and are therefore not normalized to protein concentration. The structural parameters analyzed are independent of this value (i.e., Rg, Dmax, volumes).
**http://biotools.nubic.northwestern.edu/proteincalc.html
***SAXS MoW2
****http://web.expasy.org/

Supplementary Table 3. X-ray data.

**Crystal structure**	**6HTF (rF10-SH2)**
**Data collection**	
Space group	P 21 21 2
Cell dimensions	
a, *b*, *c* (Å)	145.53, 32.95, 80.63
α, β, γ (°) (°)	90, 90, 90
Resolution (Å)	50 (2.1) *
R_{meas}	10.2 (81.1)
I / σI	12.99 (1.95)
Completeness (%)	93.93 (84.43)
Redundancy	3.89 (3.90)
**Refinement**	
Resolution (Å)	2.1
No. reflections	22064
R_{work} / R_{free}	0.213 / 0.252
No. atoms	3041
Protein	2915
Ligand/ion	0
Water	126
Protein residues	362
B-factors	
Protein	40.75
Ligand/ion	N/A
Water	36.85
R.m.s. deviations	
Bond lengths (Å)	0.023
Bond angles (°)	1.44
Ramachandran analysis	
Favored regions	95.53%
Allowed regions	4.47%
Outliers	0

*Values in parentheses are for the highest-resolution shell.

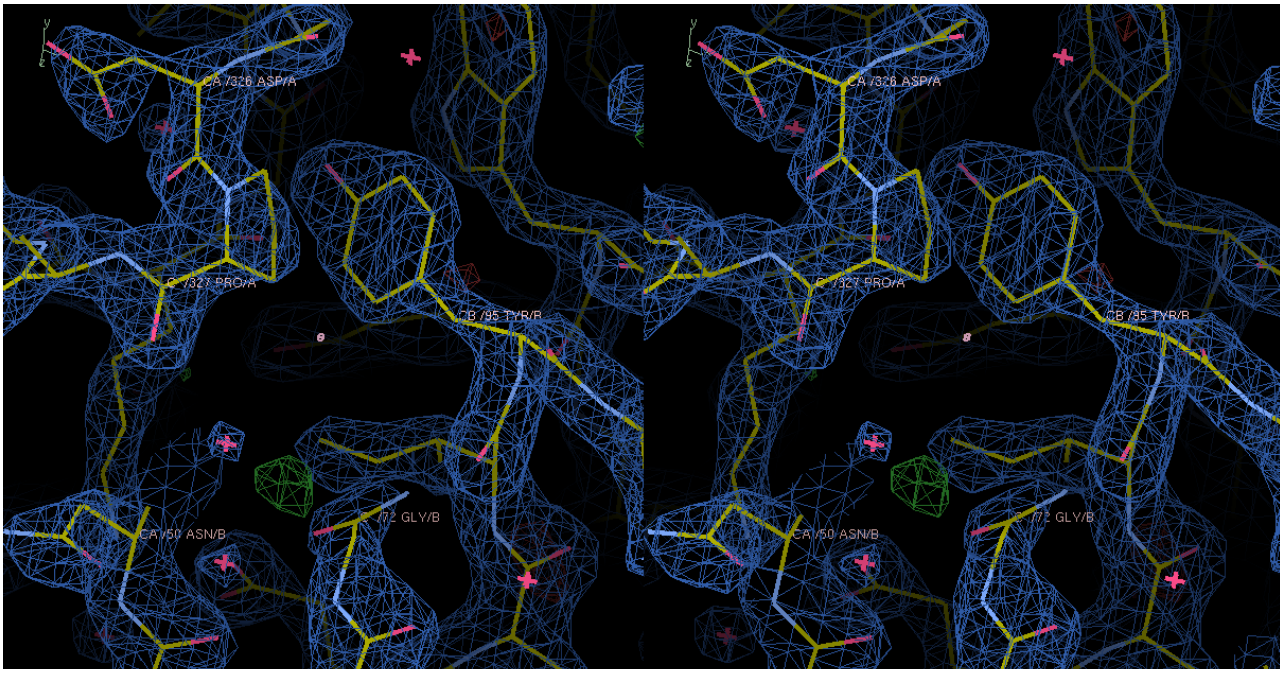

Supplementary Table 4. SAXS parameters of Btk-rF10 complexes.

**Data collection parameters**					
Instrument		MD29 beamline, ESRF Grenoble - France			
Wavelength (Å)		0.9919			
q-range (nm^{-1})		0.03563 - 5			
Exposure time (sec)		5 (10 frames x 0.5 sec)			
Temperature (K)		290			
**Samples**	**rF10**	**SH2-rF10**	**SH2-KD-rF10**	**SH3-SH2-KD-rF10**	**Full-length-rF10**
Measurement mode	SEC-SAXS	batch	SEC-SAXS	batch	batch
Concentration range ($mg.ml^{-1}$)	100 µl at 14	0.6 – 2.7	100µl at 20	0.4 – 3.6	0.4 – 2.9
SASBDB identifier	N/A	N/A	N/A	N/A	N/A
**Structural parameters**					
R_g (nm) from Guinier	2.42 ± 0.2	2.6 ± 0.3	3.46 ± 0.12	3.55 ± 0.26	4.48 ± 0.38
I(0)* (cm^{-1}) from Guinier	37.12 ± 0.06	35.7 ± 0.18	87.82 ± 0.24	61.09 ± 0.19	79.46 ± 0.23
R_g (nm) from P(r)	2.47 ± 0.006	2.6 ± 0.01	3.52 ± 0.007	3.6 ± 0.01	4.69 ± 0.02
D_{max} (nm)	7.9 ± 0.8	8.2 ± 0.8	11.5 ± 1.2	12.5 ± 1.2	16.9 ± 1.7
Porod volume (nm^3)	50.85	67.4	100.6	116.46	156.04
Dry volume calculated from sequence (nm^3)**	37.581	53.569	92.638	100.685	130.345
**Molecular mass determination (kDa)**					
From Porod volume (V_p/~1.6)	24.8	37.9	70.4	81.2	100.8
From SAXS MoW2***	27.1	41.9	79.5	86.2	116.2
Bayesian inference	28.2	37.7	67.1	74.3	94.2
From I(0) using V_c invariant	27.6	39.3	63.5	74.6	89.4
Calculated from sequence****	31.1	44.3	76.6	83.2	107.7
Number of residues	274	391	670	726	938
**Software list**					
Primary data reduction		Automated pipeline at beamline			
Data processing		PRIMUS (ATSAS v.2.8.0)			
Ab initio analysis		DAMMIN and GASBOR			
Fitting		CRYSOL			
Model refinement		SREFLEX			
Flexibility analysis		EOM 2.0			
Model superimpositions		SASpy plugin for Pymol			
3D graphics images		Pymol (v.1.8.2.1)			

*I(0) values shown in SEC-SAXS measurements vary depending on protein concentration at the analyzed peak, and are therefore not normalized to protein concentration. The structural parameters analyzed are independent of this value (i.e., Rg, Dmax, volumes).
**http://biotools.nubic.northwestern.edu/proteincalc.html
***SAXS MoW2
****http://web.expasy.org/

Supplementary Table 5. Oligonucleotides used for site-directed mutagenesis.

Primer name	Sequence 5' to 3' (forward and reverse)
Btk K296E	For: GCTGAGCAACTGCTAGAGCAAGAGGGGAAAG<br>Rev: CTTTCCCCTCTTGCTCTAGCAGTTGCTCAGC
Btk Y223F	For: GTGGCCCTTTTCGATTACATGCCAATG<br>Rev: CATTGGCATGTAATCGAAAAGGGCCAC
Btk E301K	For: GCAAGAGGGGAAAAAGGGAGGTTTCATTGTC<br>Rev: GACAATGAAACCTCCCTTTTTCCCCTCTTGC
Btk R307G	For: GGTTTCATTGTCGGCGACTCCAGCAAAGC<br>Rev: GCTTTGCTGGAGTCGCCGACAATGAAACC
Btk K311E	For: CAGAGACTCCAGCGAGGCTGGCAAATATACAG<br>Rev: CTGTATATTTGCCAGCCTCGCTGGAGTCTCTG
Btk Q341A	For: GTTGTGTGTTCCACACCTGCGAGCCAGTATTACCTGGC<br>Rev: GCCAGGTAATACTGGCTCGCAGGTGTGGAACACACAAC
Btk H364D	For: CATTAACTACCATCAGGACAACTCTGCAGGACTC<br>Rev: GAGTCCTGCAGAGTTGTCCTGATGGTAGTTAATG
Btk S371P	For: CTGCAGGACTCATACCCAGGCTCAAATATCCAG<br>Rev: CTGGATATTTGAGCCTGGGTATGAGTCCTGCAG
Btk R372G	For: CTCTGCAGGACTCATATCCGGCCTCAAATATCCAG<br>Rev: CTGGATATTTGAGGCCGGATATGAGTCCTGCAGAG
Btk K374N	For: CTCTGCAGGACTCATATCCAGGCTCAACTATCCAG<br>Rev: CTGGATAGTTGAGCCTGGATATGAGTCCTGCAGAG
Btk K400E	For: AGGTCAGGTCCTCTGGATCAATTTCCCATGATCCGT<br>Rev: ACGGATCATGGGAAATTGATCCAGAGGACCTGACCT
Btk T403K	For: CCCCAGCTCCTTCAAGAACTTCAGGTCCTTTGGATCAA<br>Rev: TTGATCCAAAGGACCTGAAGTTCTTGAAGGAGCTGGGG
Btk L405E	For: CAGTCCCCAGCTCCTTCTCGAAGGTCAGGTCCTTTG<br>Rev: CAAAGGACCTGACCTTCGAGAAGGAGCTGGGGACTG
Btk I429E	For: GCCTTCTTTGATCATCTTCTCGGCCACGTCGTACTGGCC<br>Rev: GGCCAGTACGACGTGGCCGAGAAGATGATCAAAGAAGGC
Btk C481S	For: GGTAGTTCAGGAGGCTGCCATTGGCCATGTA<br>Rev: TACATGGCCAATGGCAGCCTCCTGAACTACC
Btk D521N	For: GTTCCTTCACCGAAACCTGGCAGCTCG<br>Rev: CGAGCTGCCAGGTTTCGGTGAAGGAAC
Btk Y551F	For: GGATGATGAATTCACAAGCTCAGTAG<br>Rev: CTACTGAGCTTGTGAATTCATCATCC
