# Supplementary material for: Btk SH2-kinase interface is critical for allosteric kinase activation and its targeting inhibits B-cell neoplasms
Source: Nat Commun. 2020 May 8;11:2319. doi: 10.1038/s41467-020-16128-5 (PMC7210950; doi:10.1038/s41467-020-16128-5)
Supplement: Supplementary file 3 — Reporting Summary [file 41467_2020_16128_MOESM3_ESM.pdf]

nature research

Corresponding author(s): Oliver Hantschel

Last updated by author(s): Apr 13, 2020

Reporting Summary

Nature Research wishes to improve the reproducibility of the work that we publish. This form provides structure for consistency and transparency in reporting. For further information on Nature Research policies, see Authors & Referees and the Editorial Policy Checklist .

Statistics

For all statistical analyses, confirm that the following items are present in the figure legend, table legend, main text, or Methods section.

n/a	Confirmed	
☐	☒	The exact sample size (n) for each experimental group/condition, given as a discrete number and unit of measurement
☐	☒	A statement on whether measurements were taken from distinct samples or whether the same sample was measured repeatedly
☐	☒	The statistical test(s) used AND whether they are one- or two-sided<br>*Only common tests should be described solely by name; describe more complex techniques in the Methods section.*
☒	☐	A description of all covariates tested
☒	☐	A description of any assumptions or corrections, such as tests of normality and adjustment for multiple comparisons
☐	☒	A full description of the statistical parameters including central tendency (e.g. means) or other basic estimates (e.g. regression coefficient) AND variation (e.g. standard deviation) or associated estimates of uncertainty (e.g. confidence intervals)
☐	☒	For null hypothesis testing, the test statistic (e.g. F, t, r) with confidence intervals, effect sizes, degrees of freedom and P value noted<br>*Give P values as exact values whenever suitable.*
☒	☐	For Bayesian analysis, information on the choice of priors and Markov chain Monte Carlo settings
☒	☐	For hierarchical and complex designs, identification of the appropriate level for tests and full reporting of outcomes
☒	☐	Estimates of effect sizes (e.g. Cohen's d, Pearson's r), indicating how they were calculated

Our web collection on statistics for biologists contains articles on many of the points above.

Software and code

Policy information about availability of computer code

Data collection	Diffraction data was processed and scaled with the XDS package. Acquired SAXS data were averaged and subtracted from an appropriate solvent-blank to produce the final curve using the in-line synchrotron software and ATSAS Suite (EMBL) PRIMUS and CHROMIXS.
Data analysis	Crystallography software: molecular replacement, manual model building, B-factor refinement, solvent addition, energy-minimization and refinement of structures were performed with Phaser and Coot (Phenix version 1.13).<br>Molecular graphics: PyMOL 2.0.4 (DeLano Scientific).<br>SAXS software: PRIMUS, GNOM and CHROMIXS (final scattering curves); DAMMIF and SREFLEX (ab initio models), EOM 2.0 (flexibility of multidomain complexes) , CRYSOL (fitting of models to experimental data), SASpy (superimpositions).

For manuscripts utilizing custom algorithms or software that are central to the research but not yet described in published literature, software must be made available to editors/reviewers. We strongly encourage code deposition in a community repository (e.g. GitHub). See the Nature Research guidelines for submitting code & software for further information.

Data

Policy information about availability of data

All manuscripts must include a data availability statement. This statement should provide the following information, where applicable:

- Accession codes, unique identifiers, or web links for publicly available datasets
- A list of figures that have associated raw data
- A description of any restrictions on data availability

The X-ray structure of the rF10-SH2 complex was deposited at Protein Data Bank (entry 6HTF). Full SAXS curves and analyzed data for wild-type Btk proteins were deposited at SASBDB (entries SASDF53, SASDF63, SASDF73, and SASDF83).

Field-specific reporting

Please select the one below that is the best fit for your research. If you are not sure, read the appropriate sections before making your selection.

- [x] Life sciences
- [ ] Behavioural & social sciences
- [ ] Ecological, evolutionary & environmental sciences

For a reference copy of the document with all sections, see nature.com/documents/nr-reporting-summary-flat.pdf

Life sciences study design

All studies must disclose on these points even when the disclosure is negative.

Sample size	No sample size calculation was applied in this study. Experiments were performed in 3 biological repeats.
Data exclusions	No data was excluded.
Replication	All experiments were independently replicated as stated in the figure legends.
Randomization	NA
Blinding	No blinding, as the same investigator performed most experiments and analyzed the data.

Reporting for specific materials, systems and methods

We require information from authors about some types of materials, experimental systems and methods used in many studies. Here, indicate whether each material, system or method listed is relevant to your study. If you are not sure if a list item applies to your research, read the appropriate section before selecting a response.

Materials & experimental systems

n/a	Involved in the study	
☐	☒	Antibodies
☐	☒	Eukaryotic cell lines
☒	☐	Palaeontology
☒	☐	Animals and other organisms
☒	☐	Human research participants
☒	☐	Clinical data

Methods

n/a	Involved in the study	
☒	☐	ChIP-seq
☐	☒	Flow cytometry
☒	☐	MRI-based neuroimaging

Antibodies

Antibodies used

Immunoblotting:
Mouse monoclonal anti-Total pY (clone 4G10), dilution 1:1000, Millipore, Cat# 05-321; RRID: AB_309678
Mouse monoclonal anti-Btk (D6T2C), dilution 1:1000, Cell Signaling , Cat# 56044; RRID: AB_2799503
Rabbit polyclonal anti-Btk, dilution 1:1000, Thermo Scientific, Cat# PA5-27392; RRID: AB_2544868
Mouse monoclonal anti-Btk (pY551)/Itk (pY511) Clone 24a, dilution 1:1000, BD Biosciences, Cat# 558034; RRID: AB_2067823
Rabbit polyclonal anti-Btk (pY223), dilution 1:1000, Cell Signaling , Cat# 5082; RRID: AB_10561017
Rabbit polyclonal anti-p44/42 MAPK (Erk1/2), dilution 1:1000, Cell Signaling , Cat# 9102; RRID: AB_330744
Mouse monoclonal anti-phospho-p44/42 MAPK (Erk1/2) (Thr202/Tyr204) (E10), dilution 1:1000, Cell Signaling , Cat# 9106; RRID: AB_331768
Rabbit polyclonal anti-PLCγ2, dilution 1:1000, Cell Signaling , Cat# 3872; RRID: AB_2299586
Rabbit polyclonal anti-PLCγ2 (Tyr1217), dilution 1:1000, Cell Signaling , Cat# 3871; RRID: AB_2299548
Mouse monoclonal anti-Flag, Sigma, dilution 1:5000, Cat# F3165; RRID: AB_259529
Mouse monoclonal anti-Penta-his, dilution 1:5000, Qiagen, Cat# 34660; RRID: AB_2619735
Rabbit polyclonal anti-PLCγ2 (Tyr753) [EPR5914-3], dilution 1:1000, Abcam, Cat# ab133455; RRID: AB_2163712
Mouse monoclonal anti-Tubulin, dilution 1:2000, Sigma, Cat# T9026; RRID: AB_477593
Mouse monoclonal anti-Myc-tag Myc.A7 DyLight800, dilution 1:10,000, Thermo Scientific, Cat# MA1-21316-D800; RRID: AB_2536996
Goat polyclonal anti-mouse IgG IRDye 800CW, dilution 1:10,000, LiCor, Cat# 926-32210; RRID: AB_621842
Donkey polyclonal anti-Rabbit IgG (H+L) IRDye800, dilution 1:10,000, Rockland, Cat# 611-732-127; RRID: AB_220158
Goat polyclonal anti-Mouse IgG (H+L) Peroxidase AffiniPure, dilution 1:10,000, Jackson ImmunoResearch, Cat# 115-035-003; RRID: AB_10015289
Goat polyclonal anti-Rabbit IgG (H+L) Peroxidase AffiniPure, dilution 1:10,000, Jackson ImmunoResearch, Cat# 111-035-003; RRID: AB_2313567

Validation | All used antibodies were validated commercially and/or in previous studies.

Eukaryotic cell lines

Policy information about cell lines

Cell line source(s)	HEK293, ATCC, Cat# CRL-1573; RRID: CVCL_0045<br>HEK293T, ATCC, Cat# CRL-3216; RRID: CVCL_0063<br>Sf9 insect cells, Thermo Scientific, Cat# 11496-015<br>HBL-1, Gift from M. Thome-Miazza, RRID: CVCL_4213<br>DOHH2, Gift from M. Thome-Miazza, Cat# ACC-47; RRID: CVCL_1179<br>TMD8, Gift from M. Thome-Miazza, RRID: CVCL_A442
Authentication	Cell lines from ATCC and Thermo Scientific were authenticated by the vendors.
Mycoplasma contamination	Cell lines were regularly tested and were mycoplama negative
Commonly misidentified lines (See ICLAC register)	NA

Flow Cytometry

Plots

Confirm that:

- [x] The axis labels state the marker and fluorochrome used (e.g. CD4-FITC).
- [x] The axis scales are clearly visible. Include numbers along axes only for bottom left plot of group (a 'group' is an analysis of identical markers).
- [x] All plots are contour plots with outliers or pseudocolor plots.
- [x] A numerical value for number of cells or percentage (with statistics) is provided.

Methodology

Sample preparation	HBL-1 cells were seeded at density of 5 x 10^5 cells/well in 3 mL of cell media in 6-well cell culture plates. Cell treatments are described in Methods section. Briefly, cells were washed twice with cold PBS and resuspended in Annexin V binding buffer containing Cy5-Annexin V and 7AAD. Cells were gently dissociated, filtered through a 35 µm nylon mesh and kept on ice until FACS measurement.
Instrument	Beckman Coulter Gallios.
Software	Kaluza v1.0 for data collection and FlowJo v10.6 for data analysis.
Cell population abundance	Data from 10 x 10^4 gated events were collected for each sample.
Gating strategy	Intact cells were initially gated in a SSC/FSC area plot. Cell debris and doublets were excluded from the analysis. Unstained cells, single staining with Cy5-Annexin V or 7AAD, and double stained Annexin V/7AAD cells were used as controls for accurate gating, compensation and quadrants.

- [x] Tick this box to confirm that a figure exemplifying the gating strategy is provided in the Supplementary Information.
