# Supplementary material for: Btk SH2-kinase interface is critical for allosteric kinase activation and its targeting inhibits B-cell neoplasms
Source: Nat Commun. 2020 May 8;11:2319. doi: 10.1038/s41467-020-16128-5 (PMC7210950; doi:10.1038/s41467-020-16128-5)
Supplement: Supplementary file 4 — Description of Additional Supplementary Files [file 41467_2020_16128_MOESM4_ESM.docx]

**Description of Additional Supplementary Files**

File name: Supplementary Movie 1

Description: Superimposition of active Btk SH2-KD complex with the SH2-rF10 repebody crystal structure (6HTF). See Fig. 5h for details.
