# Supplementary material for: Btk SH2-kinase interface is critical for allosteric kinase activation and its targeting inhibits B-cell neoplasms
Source: Nat Commun. 2020 May 8;11:2319. doi: 10.1038/s41467-020-16128-5 (PMC7210950; doi:10.1038/s41467-020-16128-5)
Supplement: Supplementary file 2 — Peer Review File [file 41467_2020_16128_MOESM2_ESM.pdf]

Reviewers' comments:

Reviewer #1 (Remarks to the Author):

Dear Nature Communications,

The manuscript entitled “Btk SH2-kinase interface is critical for allosteric kinase activation and its targeting inhibits B-cell neoplasms” by Duarte and colleagues describes the finding of an allosteric site on the kinase Btk that is required for it to become activated and the development of a repebody to bind to that region. Btk is the target of the drug ibrutinib which is a widely used and effective treatment for B-cell cancers and is therefore of significant pharmacological interest. Although ibrutinib is highly effective, resistance to therapy can arise by mutation of the kinase and therefore different modes of targeting the molecule are of interest. The identification of an allosteric site in this manuscript could aid such an approach.

The authors describe some MD simulations which suggest a loose association with the back-side of the SH2 domain and the N-lobe of the kinase. This side of the SH2 domain is the site of several disease-causing mutations in BTK which suggests a functional consequence. These mutations lead to a loss of kinase activation whilst they do not impair phosphopeptide binding. SAXS analysis by the authors suggests that the kinase can exist in an extended conformation and surmise that this is necessary for activation. Finally they develop a protein that binds to the allosteric site and show in cells and in vitro that it can inhibit autoactivation. This suggests that this site may be a genuine target for drug development

This is a very detailed manuscript with an extremely impressive amount of experimental data. Although there is no “killer” experiment that proves the existence of this allosteric site there is a sufficient quantity of supporting data to imply that it exists. Of course, the existence of an allosteric site would be a lot more convincing if there were mutations in the predicted site on the N-lobe of the kinase that were made that interfered with activation, however that could prove to be a very difficult undertaking and I don’t suggest that it be required for publication. My recommendation is that the manuscript is of sufficient interest and novelty to be published in Nature Communications.

Comments:

Results: line 132: The authors state that stability is unaffected however in Supp Fig 1 it is clear that 4 of the 5 mutants have a decreased thermal stability of 5-6 degrees which is actually quite significant for a single point mutation.

Results: line 222: I’m not sure what this sentence means. Do the authors mean that the linker was removed and MD performed on the SH2 and KD domains as separate chains? If so, it is not surprising that the interaction didn’t occur as tethering of two domains vastly increases their chance of interacting and does not point to individual residues in the linker interacting directly.

Results: line 237: “excellent agreement” is an overstatement for the sh3-sh2-kd data

Results: line 256: Both K296E and S371P are disease causing mutations in Btk however they have opposite effects on autophosphorylation-do the authors have an explanation for this?

Results: line 337: The interpretation of the SAXS data indicating a disruption in the interdomain interface is very tenuous. The SAXS-based reconstructions are of too low a resolution to “indicate” such a thing, maybe “suggest” is a more appropriate verb.

Results: Line 344: This is an important result as it shows that the rF10 site is not "hidden" in longer forms of the protein – have the authors performed the ITC experiment more than once?

Results: line 366: Was this with btk that was pre-auto-activated? If so its quite a surprising result.

Results: line 396: "..rF10 was able to decrease pY551 of different C481S constructs". Is that true? Figure 7b says only the SH2-KD phosphorylation was significantly inhibited

Minor comments:

Intro: line 83: How common is resistance to ibrutinib?

Reviewer #2 (Remarks to the Author):

This manuscript describes the use of a battery of methods and a set of mutants to decipher the domain interaction in BTK and suggests that peptides may be used to interfere with the SH2 and kinase domains, including serving as inhibitors for this kinase. It also contains a novel structure for the BTK SH2 domain.

The first part is clear although there are certain issues deserving comments:

1. In the ABSTRACT line 30, it is stated "…constitutive BTK signaling drives several B-cell neoplasms…". For tumors responding to BTK inhibitors (BTKi), only rarely is constitutive BTK signaling a driver; rather tumor cells, similar to normal B-cells, are addicted to BTK for the adhesive processes necessary for these cells to remain in lymphoid organs, where they thrive.

2. In the INTRODUCTION, line 76 & continued, I do not find that the history of BTKi development is correctly described. As an example, the BTKi, which later was named ibrutinib, was developed early on and published already in 2007 by Pan Z et al., and this paper is not cited.

3. Fig. 1f. BTK WT's relative pY551 could be 100 or 1 rather than around 75.

4. Line 325, the fold increase seems more like ~6.5 rather than ~10.

The second part, aiming at demonstrating that repebody protein, rF10, works as a BTKi, is more problematic, although from Fig. 6, panel a, rF10-induced expression seems to negatively influence the survival of HBL-1 cells.

The basis for BTK activation is that SRC-family kinases like LYN phosphorylates BTK at Y551 (the classical initial activation step for all cytoplasmic protein-tyrosine kinases) and subsequently the main BTK substrate PLCG2 is phosphorylated. Since BTK also can be its own substrate, pY223 is frequently used as a measure of BTK's catalytic activity. When inhibitors are used it is the effect on BTK Y223 and PLCG2, which is the predominant readout. The authors of this manuscript instead seem to concentrate on the effect on pY551 (e.g. Fig. 6 d). The pY223 level is severely lowered by ibrutinib for the HBL-1 cell line in Fig. 6, panel c (this control is missing from the DOHH2 cells in Fig. 6, panel e), whereas rF10 seems to have a more modest effect and the same is true also for the PLCG2 substrate, as measured by pY1217 and pY753.

ERK phosphorylation, which is a downstream in the B-cell receptor signaling pathway and representing an indirect measure of BTK enzyme function, seems essentially unaffected in Fig. 6, panel c, whereas a very potent effect is noticed in Fig. 6, panel e (i.e. in spite of rather weak activity on BTK Y223 and pY753/1217 in PLCG2, vide supra). This result could be interpreted as if rF10 has an effect on the

phosphorylation of ERK in DOHH2 cells, which is BTK-independent; moreover, such an effect was not obvious in the HBL-1 cells. Collectively, while the authors claim effects on BTK pY223 and PLCG2 pY753/pY1217 these effects are not as visible as the one on BTK pY551.

To complement these figures there is also supplementary material. BTK inhibition can impair survival and cell division of sensitive target B-cells. This is stated by the authors, but from e.g. Supplementary Fig. 6 b this is not obvious – is it a single measurement which is statistically different?

A possible way to study this would be to combine e.g. ibrutinib treatment and rF10-induced expression in some form of titration experiment.

Minor points:
Line 161 resembles (not resemble)
Line 466 provides (not provide)
Line 490 DNase (not DNAse)
Line 715 Dawn Heleos (not Dawan Hellios)

Some sentences need improvement/correction

Reviewer #3 (Remarks to the Author):

In this manuscript, Duarte et al. provide biophysical and biochemical evidence that the SH2 domain of Btk plays a critical role in the activation of its kinase domain, and that a subset of loss-of-function Btk mutations in the SH2 domain that have been linked to human X-linked agammaglobulinemia (XLA) are due to loss of this role. In addition, the authors have developed a repebody (LRR protein) that binds with high affinity to the Btk SH2 domain and inhibits Btk kinase activity in vitro and in cells, evidently by abrogating the stimulatory SH2-kinase interaction. A crystal structure of the repebody with the SH2 domain was determined to understand the precise binding mode. The repebody was tested in vitro and in several cell lines and found to inhibit Btk autophosphorylation and signaling. This study will be of strong interest to those in the protein kinase and kinase therapeutics fields.

Major concern:

• A major aspect of this story is that the Btk SH2 domain binds to the N lobe of the kinase domain to stimulate tyrosine kinase activity, yet the authors tested only one mutation in the SH2 domain, S371P (an XLA mutation), to probe the putative SH2-kinase interface suggested by the MD simulations and SAXS data. Other mutations in the putative interface should be tested, irrespective of whether they are XLA mutations. Better yet would be the design of a gain-of-function point mutation in the SH2 domain (in the putative interface), which leads to increased phosphorylation of Y551 relative to wild-type. This should be possible, given that the SH2-kinase stimulatory interaction appears to be quasi-stable, i.e., there’s room for improvement.

Minor point:
• The Introduction (first part) could be shortened; a review of protein kinases as targets of small-molecule inhibitors is not necessary.

Point-by-point response to the reviewers' comments

Our comments in red, **additional data/changes to manuscript/figures in bold red**

Reviewer #1 (Remarks to the Author):
Dear Nature Communications,
The manuscript entitled "Btk SH2-kinase interface is critical for allosteric kinase activation and its targeting inhibits B-cell neoplasms" by Duarte and colleagues describes the finding of an allosteric site on the kinase Btk that is required for it to become activated and the development of a repebody to bind to that region. Btk is the target of the drug ibrutinib which is a widely used and effective treatment for B-cell cancers and is therefore of significant pharmacological interest. Although ibrutinib is highly effective, resistance to therapy can arise by mutation of the kinase and therefore different modes of targeting the molecule are of interest. The identification of an allosteric site in this manuscript could aid such an approach.
The authors describe some MD simulations which suggest a loose association with the back-side of the SH2 domain and the N-lobe of the kinase. This side of the SH2 domain is the site of several disease-causing mutations in BTK which suggests a functional consequence. These mutations lead to a loss of kinase activation whilst they do not impair phosphopeptide binding. SAXS analysis by the authors suggests that the kinase can exist in an extended conformation and surmise that this is necessary for activation. Finally they develop a protein that binds to the allosteric site and show in cells and in vitro that it can inhibit autoactivation. This suggests that this site may be a genuine target for drug development
This is a very detailed manuscript with an extremely impressive amount of experimental data. Although there is no "killer" experiment that proves the existence of this allosteric site there is a sufficient quantity of supporting data to imply that it exists. Of course, the existence of an allosteric site would be a lot more convincing if there were mutations in the predicted site on the N-lobe of the kinase that were made that interfered with activation, however that could prove to be a very difficult undertaking and I don't suggest that it be required for publication. My recommendation is that the manuscript is of sufficient interest and novelty to be published in Nature Communications.

> We thank the reviewer for her/his very positive comments on our manuscript and valuing the amount of data and level of detail of the manuscript.
> As further mutational analysis of the allosteric SH2-kinase interface was also requested by reviewer 3, we have tested point mutations in the N-lobe of the kinase domain that were structurally predicted to be critical for Btk allosteric activation. We show that these mutations impair Btk activation complementing the data on mutations in the SH2 domain. **The additional data is shown in SI Fig. 3k-m and mentioned in the text (lines 241-247).**
> Furthermore, as detailed in our response to reviewer 3, we could identify a point mutation in the N-lobe (L405E) that resulted in a very strong over-activation of Btk, compatible with a gain-of-function mutation that would stabilize the SH2-kinase interface by establishing an additional salt bridge with positively charges residues in the SH2 domain **(SI Fig. 3k-m).**

Comments:
Results: line 132: The authors state that stability is unaffected however in Supp Fig 1 it is clear that 4 of the 5 mutants have a decreased thermal stability of 5-6 degrees which is actually quite significant for a single point mutation.

> We agree with reviewer that the Tm of some mutant SH2 domains (e.g. K296E) is ~5 degrees lower than the Tm for the WT SH2 domain. However, the Tm for the SH2 mutants is still high (all above 47˚C) when measured in the SH2 domain alone, and

under physiological conditions in the full-length protein context will likely have an even lower impact. Additionally, when interpreting the complete physicochemical characterization performed for these mutant domains (SEC, CD, melting, pY-peptide binding), we observed that all the SH2 domains behave very well as recombinant proteins and remain fully folded and functional.
We have revised the statement in the text that a decrease in Tm was observed (line 125).

Results: line 222: I'm not sure what this sentence means. Do the authors mean that the linker was removed and MD performed on the SH2 and KD domains as separate chains? If so, it is not surprising that the interaction didn't occur as tethering of two domains vastly increases their chance of interacting and does not point to individual residues in the linker interacting directly.

As pointed out by the reviewer, it is not surprising that MD performed on the SH2 and KD domains as separate chains did not lead to an interaction. This was also our expectation, but we still performed these simulations, which confirmed this. The MD simulations that we performed then were indeed performed including the (native) SH2-KD linker region sequence as described in detail in the Methods section. **To avoid confusion, we have removed the misleading sentence and have revised the paragraph to make clear that the SH2-kinase unit including its native linker were used for the MD (line 202-203).**

Results: line 237: "excellent agreement" is an overstatement for the sh3-sh2-kd data

We have revised our original statement. ("*Ab initio* shape reconstructions from SAXS corroborate the available KD and (closed autoinhibited) SH3-SH2-KD crystal structures.": line 230)

Results: line 256: Both K296E and S371P are disease causing mutations in Btk however they have opposite effects on autophosphorylation-do the authors have an explanation for this?

This is indeed an interesting point. XLA mutations can affect Btk function in various ways, which include decreased protein stability, changed protein subcellular localization, effects on catalytic activity, or impairment of Btk interactions/scaffolding and signaling in B cells. Our *in vitro* autophosphorylation assays demonstrated that S371P has a negative impact on the protein phosphorylation capacity, while K296E does not. This suggests that K296E, which is not facing the predicted interface with the KD, causes XLA by another mechanism than affecting the SH2-KD allosteric interaction. This is the first study exploring the XLA mutation K296E. It is important to remember that we selected K296E as a control mutation, which is not facing the predicted site of interaction with the kinase N-lobe. Therefore, another mechanism than perturbation of SH2-kinase interaction must be responsible for the loss-of-function phenotype of this XLA mutation, which must be subject of an independent study.

Results: line 337: The interpretation of the SAXS data indicating a disruption in the interdomain interface is very tenuous. The SAXS-based reconstructions are of too low a resolution to "indicate" such a thing, maybe "suggest" is a more appropriate verb.

We have revised the text, as suggested (line 335).
Still, we would like to point out that we draw this conclusion based on structural information coming from multiple sources, which include the active conformation found by the MD simulation, superimposition of crystal structures of SH2-repebody complex,

and extensive SAXS modelling against experimental scattering data (Btk WT SH2-KD shown in SI Fig. 4D, as well with kinase-dead protein). We believe that the combination of these structural datasets provides us enough indication that the active Btk complex (i.e. SH2-KD) is somewhat disrupted upon binding of the repebody. We are aware that SAXS provides a low-resolution model and also shows certain flexibility (see Kratky plot in SI Fig. 4D). Hence, we have included this possible solution of the SH2-KD-repebody complex to the supplementary material only. Finally, although the chi^2 is higher for this superimposition (Chi^2=3.1), it shows an acceptable visual agreement with the SAXS experimental curve measured using CRYSOL (included below).

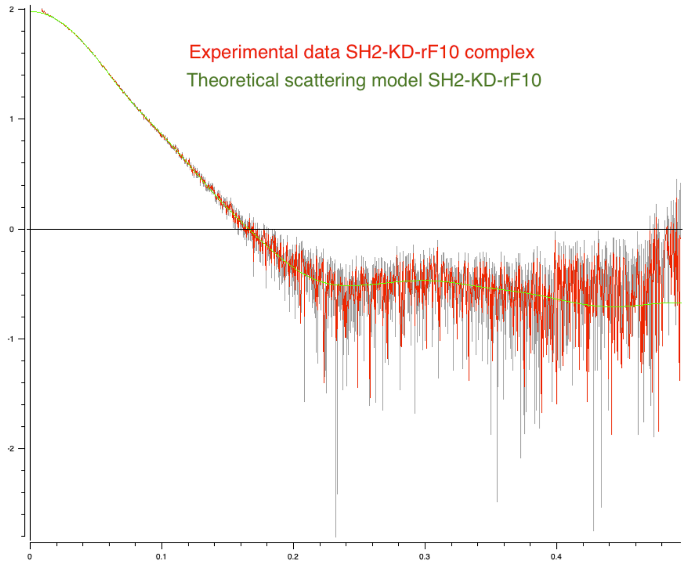

Results: Line 344: This is an important result as it shows that the rF10 site is not “hidden” in longer forms of the protein – have the authors performed the ITC experiment more than once?

These ITC experiments with the SH2-KD, SH3-SH2-KD and full-length Btk were done twice for each protein. There was excellent agreement between replicates and the mean Kd ± SD calculated from both replicates are displayed along with one representative ITC curves in SI Fig. 5A.
Noteworthy, superimpositions of autoinhibited FL and SH3-SH2-KD with the crystal structure from SH2-rF10 indicates that the site of binding to the rF10 is exposed and able to bind even in these closed conformations (see example in SH3-SH2-KD-rF10 model from SAXS in SI Fig 4D).

Results: line 366: Was this with btk that was pre-auto-activated? If so its quite a surprising result.

The recombinant Btk preparations that we used displayed strong *in vitro* kinase activity in line with the results in Fig. 2c. There was no pre-activation step performed in this experiment, only addition of the repebody prior to the kinase assay reaction.
In general, overexpression of full-length cytoplasmic kinases in Sf9 cells (or other heterologous expression hosts) may partly disrupt autoinhibition, which results in higher kinase activity, which can then be inhibited e.g. with a repebody. This is a common observation that we and many others made for Abl and Src kinases already 20-30 years ago, and also in lines with what we and others observed for Btk. This may be one reason why the autoinhibited full-length Btk has been resilient to crystallization (as are other full-length cytoplasmic TKs, such as Abl).

Results: line 396: "..rF10 was able to decrease pY551 of different C481S constructs". Is that true? Figure 7b says only the SH2-KD phosphorylation was significantly inhibited

> **We have revised the text and apologize for the misleading statement (line 401-402).** Indeed HEK transfections with mutants show a significant decrease only for SH2-KD C481S, which is the most active construct. The mutant SH3-SH2-KD and FL proteins show low pY551 levels to begin with and did not show significant inhibition when co-expressed with the repebody in HEK cells.

Minor comments:

Intro: line 83: How common is resistance to ibrutinib?

> About 5% of CLL patients treated with ibrutinib progress and acquire resistance to the drug. Among these, mutations on Btk C481 and/or PLCG2 are found in about 80% of the refractory patients (Lampson & Brown (2018) Expert Rev Hematol. 11(3):185-194). Another recent study from France reported that Btk mutations were found in about a half (57%) of patients receiving ibrutinib with/without signs of disease progression, while PLCG2 mutations were less frequent (13%) (Quinquenel et al (2019) Blood 134, 641-644).

Reviewer #2 (Remarks to the Author):

This manuscript describes the use of a battery of methods and a set of mutants to decipher the domain interaction in BTK and suggests that peptides may be used to interfere with the SH2 and kinase domains, including serving as inhibitors for this kinase. It also contains a novel structure for the BTK SH2 domain.

The first part is clear although there are certain issues deserving comments:

1. In the ABSTRACT line 30, it is stated "…constitutive BTK signaling drives several B-cell neoplasms…". For tumors responding to BTK inhibitors (BTKi), only rarely is constitutive BTK signaling a driver; rather tumor cells, similar to normal B-cells, are addicted to BTK for the adhesive processes necessary for these cells to remain in lymphoid organs, where they thrive.

> The remark of the reviewer is of course correct and the necessary brevity of the abstract resulted in this misleading statement. **We have revised the sentence to the following: "...Btk signaling sustains growth of several B-cell neoplasms, which may be treated with tyrosine kinase inhibitors (TKIs)." (line 30)**

2. In the INTRODUCTION, line 76 & continued, I do not find that the history of BTKi development is correctly described. As an example, the BTKi, which later was named ibrutinib, was developed early on and published already in 2007 by Pan Z et al., and this paper is not cited.

> **We have added the references and rephrased the paragraph (line 70-78).** We thank the reviewer for the suggestion.

3. Fig. 1f. BTK WT's relative pY551 could be 100 or 1 rather than around 75.

We have now normalized the data to 100, as requested.

4. Line 325, the fold increase seems more like ~6.5 rather than ~10.

The fold-difference in affinity is 8.7-fold (15.5nM for WT vs. 135nM for the K374N mutant). **We have corrected the statement in text to ~9-fold (line 325).**

The second part, aiming at demonstrating that repebody protein, rF10, works as a BTKi, is more problematic, although from Fig. 6, panel a, rF10-induced expression seems to negatively influence the survival of HBL-1 cells.

The basis for BTK activation is that SRC-family kinases like LYN phosphorylates BTK at Y551 (the classical initial activation step for all cytoplasmic protein-tyrosine kinases) and subsequently the main BTK substrate PLCG2 is phosphorylated. Since BTK also can be its own substrate, pY223 is frequently used as a measure of BTK's catalytic activity. When inhibitors are used it is the effect on BTK Y223 and PLCG2, which is the predominant readout.

From our prior (albeit limited) contributions to the Btk field, we obtained the impression that the reasons for pY223 being the more common read-out for Btk activation are not biological, but rather technical/historical. Firstly, most pY223 antibodies were historically more commonly used due to their better sensitivity and reliability, as opposed to pY551 antibodies, which furthermore only became commercially available from different suppliers ~10 years ago. Secondly, as mentioned by this reviewer, for inhibitor studies BTK Y223 and PLCG2 are the predominant readouts. But the reason for this is the paradoxical increase of pY551 (despite inhibition of kinase activity) upon treatment with Btk inhibitors that induce an open conformation of the activation loop. Therefore, in this context, pY551 is indeed not a suitable readout. The observation of paradoxical activation loop phosphorylation was nicely demonstrated with type 1 JAK TKIs (see. Andraos, ..., Radimerski 2012 Cancer Discovery), but also for other kinases/type I TKIs and also seen in this study (Fig. 6C and 6E, third lane from left). Nevertheless, Btk Y551 in the activation loop is a prime site for kinase activation, whose phosphorylation we have assayed along with pY223.

The authors of this manuscript instead seem to concentrate on the effect on pY551 (e.g. Fig. 6 d). The pY223 level is severely lowered by ibrutinib for the HBL-1 cell line in Fig. 6, panel c (this control is missing from the DOHH2 cells in Fig. 6, panel e), whereas rF10 seems to have a more modest effect and the same is true also for the PLCG2 substrate, as measured by pY1217 and pY753.

Please note that ibrutinib is included as control also for the DOHH2 cells in Fig. 6E. We also want to mention that pY223 cannot be universally used for our study, as Y223 is located in the SH3 domain. Therefore, it is not available to study activation of the SH2-KD construct, which has the highest activity and the one we focused on to understand the molecular basis of the SH2-XLA mutations before we moved on to constructs including the SH3 domain (and pY223) and to malignant cells.

Our experiments show that expression of the rF10 repebody in B cells decrease pY551, but seems to have a lower impact on the pY223, which at first sight might seem incoherent. But, as demonstrated for Abl and Fes kinases, the SH2-kinase interface is also critical for kinase substrate recruitment and (processive) multi-site phosphorylation. Therefore, it is conceivable that auto-phosphorylation of Btk pY223 is only mildly affected by targeting the SH2-kinase interface, whereas trans-phosphorylation of substrates (and trans-autophosphorylation of Y551) is strongly perturbed and results in induction of apoptosis of Btk-dependent cells.
Thus, we believe that the clear effect on Y551 and downstream signalling is sufficient to demonstrate that targeting solely the SH2-KD interface is a more suitable marker of Btk activity than pY223.

ERK phosphorylation, which is a downstream in the B-cell receptor signaling pathway and representing an indirect measure of BTK enzyme function, seems essentially unaffected in Fig. 6, panel c, whereas a very potent effect is noticed in Fig. 6, panel e (i.e. in spite of rather weak activity on BTK Y223 and pY753/1217 in PLCG2, vide supra). This result could be interpreted as if rF10 has an effect on the phosphorylation of ERK in DOHH2 cells, which is BTK-independent; moreover, such an effect was not obvious in the HBL-1 cells. Collectively, while the authors claim effects on BTK pY223 and PLCG2 pY753/pY1217 these effects are not as visible as the one on BTK pY551.

We were/are also puzzled about some of these observations between different cell lines and an easy explanation may not exist: Firstly, there is a great genetic heterogeneity among the different B cell lymphoma cell lines tested with different Btk expression and activation states, different degree of IgM/G stimulatibility, expression/activation of downstream signaling effectors, which all result in wiring differences of the BCR signaling networks in each cell line. Secondly, we observed different repebody expression levels in the cell lines, which may suffice in one cell line to inhibit critical pathways resulting in apoptosis, but not in another cell line. Thirdly, as allosteric targeting the SH2-kinase interface is a pharmacologically very different process than blocking the ATP-binding site with ibrutinib, a less global and more diverse response is not unexpected. Lastly, we cannot rule out that Btk may have downstream effectors in HBL-1 other than ERK that play a critical functional role for survival/sustained proliferation. In line with this argument, even with ibrutinib, we did not observe a decrease of pERK (Fig. 6C, 3rd lane from left) despite growth inhibition with ibrutinib! Vice versa, in DOHH2, pErk is strongly inhibited by either ibrutinib or expression of the rF10 repebody, but these cells are only sensitive to ibrutinib.
Collectively, although we do not have a full explanation of all effects of the repebody on Btk signaling and do not have a biomarker to predict sensitivity of cell lines, we demonstrate inhibition of cell proliferation in two of three tested cell lines, concomitant with inhibition of Btk pY551 and other BCR signaling effectors, which is in line with an on-target effect of the repebody and indicating a functional relevance of the SH2-kinase interface for proliferation/survival.

To complement these figures there is also supplementary material. BTK inhibition can impair survival and cell division of sensitive target B-cells. This is stated by the authors, but from e.g. Supplementary Fig. 6 b this is not obvious – is it a single measurement which is statistically different?

All cell proliferation assays (including SI Fig. 6B) were performed in two technical replicates for each of the three independent biological assays (n=3). Note that the plots in Fig 6A and SI Fig. 6B display the cumulative cell numbers on a log10 scale. We

detected significant changes in HBL-1 and TMD8 upon the expression of rF10, and different from all controls included in the experiment (parental cells, all uninduced controls and control repebody ± DOX). The smaller relative inhibition of cell growth by the repebody in TMD8, as opposed to HBL-1 cells, might be the lower expression level of the repebody that we could achieve in TMD8 cells.

A possible way to study this would be to combine e.g. ibrutinib treatment and rF10-induced expression in some form of titration experiment.

As suggested, we have measured dose-response curves for ibrutinib in the presence of rF10 or control repebody for HBL-1 cells: Upon allosteric inhibition of the SH2-kinase interface with the rF10 repebody, Btk remains as sensitive to ibrutinib inhibition as the unperturbed protein. Furthermore, combined treatment with ibrutinib and repebody further increases levels of apoptosis, even when compared to the strong apoptosis induced by ibrutinib alone. **This additional data is shown in SI Fig. 6D and E, and we have added two sentences to the results text (lines 381-386).** This argues that there might a benefit in targeting concomitant or sequential with Btk TKIs and probes that interfere with allosteric regulation of the SH2-kinase domain interface.

Minor points:
Line 161 resembles (not resemble)
Line 466 provides (not provide)
Line 490 DNase (not DNAse)
Line 715 Dawn Heleos (not Dawan Hellios)

Some sentences need improvement/correction

Spelling mistakes were corrected as suggested and the manuscript was checked for spelling/grammar mistakes.

Reviewer #3 (Remarks to the Author):

In this manuscript, Duarte et al. provide biophysical and biochemical evidence that the SH2 domain of Btk plays a critical role in the activation of its kinase domain, and that a subset of loss-of-function Btk mutations in the SH2 domain that have been linked to human X-linked agammaglobulinemia (XLA) are due to loss of this role. In addition, the authors have developed a repebody (LRR protein) that binds with high affinity to the Btk SH2 domain and inhibits Btk kinase activity in vitro and in cells, evidently by abrogating the stimulatory SH2-kinase interaction. A crystal structure of the repebody with the SH2 domain was determined to understand the precise binding mode. The repebody was tested in vitro and in several cell lines and found to inhibit Btk autophosphorylation and signaling. This study will be of strong interest to those in the protein kinase and kinase therapeutics fields.

We thank the reviewer for her/his encouraging comments on our manuscript and recognizing its value for the protein kinase field.

Major concern:

• A major aspect of this story is that the Btk SH2 domain binds to the N lobe of the kinase domain to stimulate tyrosine kinase activity, yet the authors tested only one mutation in the SH2 domain, S371P (an XLA mutation), to probe the putative SH2-kinase interface suggested by the MD simulations and SAXS data. Other mutations in the putative interface should be

tested, irrespective of whether they are XLA mutations. Better yet would be the design of a gain-of-function point mutation in the SH2 domain (in the putative interface), which leads to increased phosphorylation of Y551 relative to wild-type. This should be possible, given that the SH2-kinase stimulatory interaction appears to be quasi-stable, i.e., there's room for improvement.

Following these suggestions, we have tested point mutations in the N-lobe of the kinase domain that were structurally predicted to be critical for Btk allosteric activation. We show that several of these mutations impair Btk activation complementing the data on mutations in the SH2 domain. **The additional data is shown in SI Fig. 3K-N and mentioned in the text (lines 241-147).**
Additional XLA and non-XLA mutations in the SH2 domain were tested. The great majority resulted on lower activation are shown below for the reviewers and editor. Given that we already include data on more than a dozen SH2 mutations in the manuscript, we think that inclusion of this dataset would not add additional insight, but rather confuse readers

Concerning the design of gain-of-function point mutations, we took this suggestion seriously despite the significant conceptual and technical challenges.
Conceptual challenges: Based on our data, the SH2-KD construct is already super-active, as it lacks autoinhibitory constraints plus is allosterically activated by the SH2-KD interaction, giving little room for further increases in activity.
Technical challenges: There is very little evidence that tyrosine kinase gain-of-function mutations can be rationally engineered based on structural data. Either selection of mutation from a large combinatorial library using e.g. yeast display could be attempted, which is beyond the scope of this paper. The only case of a gain-of-funtion mutation of an already activated kinase that we are aware of, is our own work on the T231R gain-of-function mutation in the Abl kinase. But one should keep in mind that this mutation was identified from a cohort of >100 imatinib-resistant CML patients and only retrospectively been shown to act by establishing an additional salt bridge between the SH2 and kinase domains (see: Sherbenou et al. 2010 Blood and Lamontanara et al. 2014 Nat. Comm.)
Still, given these challenges, we could identify a point mutation in the N-lobe (L405E) that resulted in a very strong over-activation of Btk SH2-KD! Due to the structural location of this mutation in the N-lobe, it is unlikely that it would intrinsically activate the kinase domain, while our observation is compatible with a gain-of-function caused by stabilization of the SH2-kinase interface. A possible explanation is the establishment of an additional salt bridge with positively charges residues in the SH2 domain.
This additional data and a structural model are included in SI Fig. 3K-N and mentioned in the manuscript text (line 243-247).

Additional XLA mutation in the SH2 domain:

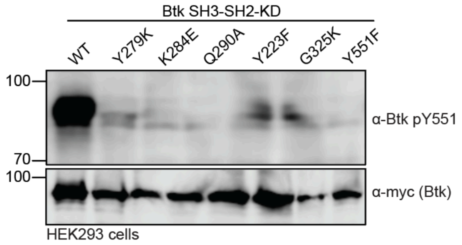

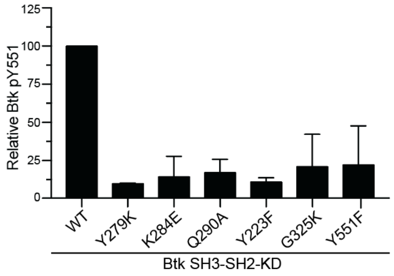

Additional non-XLA mutations in the SH2 domain:

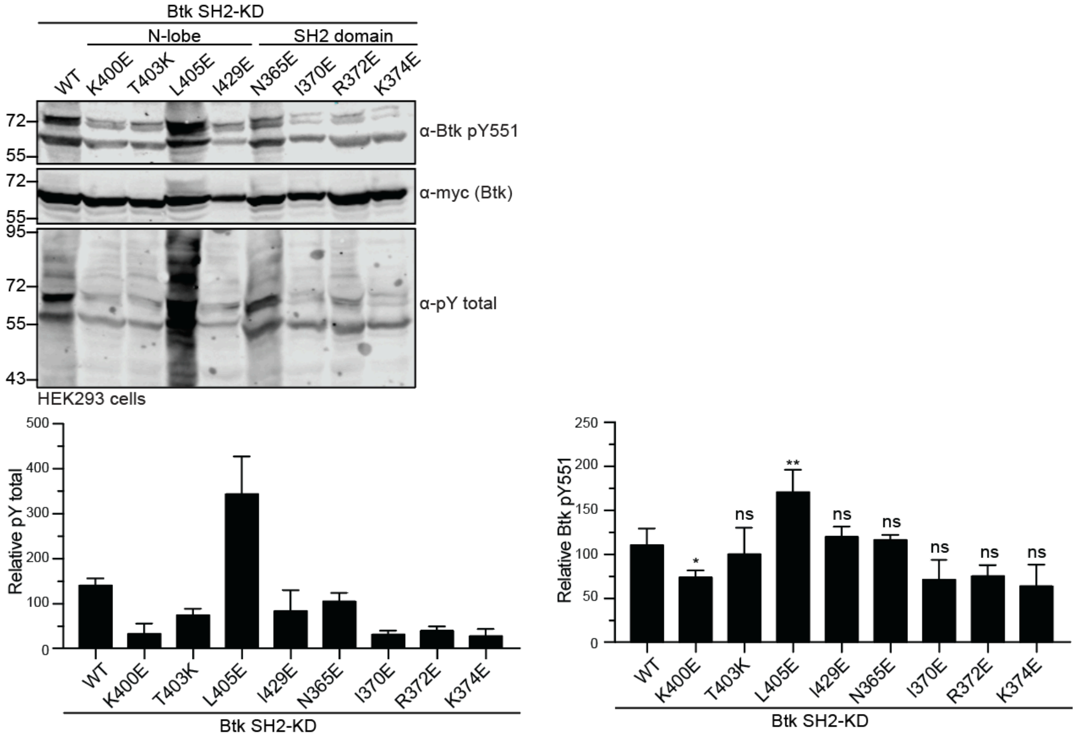

Minor point:

• The Introduction (first part) could be shortened; a review of protein kinases as targets of small-molecule inhibitors is not necessary.

We have shortened the first part of the introduction mildly, but, as *Nature Communications* is a journal with a broad readership, a more extensive introduction on protein kinases as drug targets is warranted than in a more specialized journal focusing on signaling or pharmacology/drug discovery.

REVIEWERS' COMMENTS:

Reviewer #1 (Remarks to the Author):

All of my queries/concerns have been adequately dealt with

Reviewer #2 (Remarks to the Author):

The authors have satisfactorily answered most of my queries. I still have some concerns regarding the effect on phosphorylation on Y551 versus Y223 in BTK and the phosphorylation of the substrate PLCG2. However, given that the manuscript describes a novel mechanism of regulation for BTK, it seems unreasonable to demand full explanation for every measurement and observation. The manuscript contains many interesting features, apart from what was already mentioned, also new data on the structure of BTK.

C. I. Edvard SMITH

Reviewer #3 (Remarks to the Author):

The authors have provided additional mutagenesis data supporting the SH2 domain-kinase N-lobe interaction that stimulates catalytic activity, although it is not surprising that a mutation such as I429E, which lies in the 'hydrophobic core' of the N lobe, disrupts kinase activity, probably because it disrupts intrinsic catalytic activity, not because the allosteric interaction with the SH2 domain is lost (or at least not exclusively).

The authors suggest that L405E in the kinase N lobe might hyperactivate the kinase domain by hyperstabilizing the interaction with the SH2 domain. They mention in the text the possible interaction between L405E and positively charged residues in the SH2 domain, citing Suppl. Fig. 3n, yet this figure does not show the location of the positively charged residues. The authors might want to modify this figure to do so.

Point-by-point response to the reviewer's comments
Our comments in red, **additional data/changes to manuscript/figures in bold red**

REVIEWERS' COMMENTS:

Reviewer #1 (Remarks to the Author):

All of my queries/concerns have been adequately dealt with.

We thank you the reviewer for her/his help in improving this manuscript.

Reviewer #2 (Remarks to the Author):

The authors have satisfactorily answered most of my queries. I still have some concerns regarding the effect on phosphorylation on Y551 versus Y223 in BTK and the phosphorylation of the substrate PLCG2. However, given that the manuscript describes a novel mechanism of regulation for BTK, it seems unreasonable to demand full explanation for every measurement and observation. The manuscript contains many interesting features, apart from what was already mentioned, also new data on the structure of BTK.
C. I. Edvard SMITH

We are delighted that Prof. Smith served as a reviewer of our manuscript and about his positive comments.

Reviewer #3 (Remarks to the Author):

The authors have provided additional mutagenesis data supporting the SH2 domain-kinase N-lobe interaction that stimulates catalytic activity, although it is not surprising that a mutation such as I429E, which lies in the 'hydrophobic core' of the N lobe, disrupts kinase activity, probably because it disrupts intrinsic catalytic activity, not because the allosteric interaction with the SH2 domain is lost (or at least not exclusively).
The authors suggest that L405E in the kinase N lobe might hyperactivate the kinase domain by hyperstabilizing the interaction with the SH2 domain. They mention in the text the possible interaction between L405E and positively charged residues in the SH2 domain, citing Suppl. Fig. 3n, yet this figure does not show the location of the positively charged residues. The authors might want to modify this figure to do so.

Thank you for pointing this out. Indeed, we cannot exclude that N-lobe mutations may disrupt the activity intrinsic to the kinase domain, such as the I429E mutation. This is the reason why we provided data for several new non-XLA mutation in the N-lobe. Supplementary Figure 3n (now Supplementary Figure 3f) was modified, as suggested, to allow a better overview of the position of residue L405 and corresponding positively changed residues on the SH2 domain.
We thank you the reviewer for her/his help in improving this manuscript.
